# Sepsis alters NK cell transcriptional programs for stress, actin remodeling, and intracellular trafficking

**DOI:** 10.1186/s11658-025-00851-2

**Published:** 2026-02-04

**Authors:** Holger A. Lindner, Carolina de la Torre, Sonia Y. Velásquez, Jutta Schulte, Carsten Sticht, Manfred Thiel, Anna Coulibaly

**Affiliations:** 1 Department of Anesthesiology, Surgical Intensive Care Medicine and Pain Medicine, Mannheim Institute for Innate Immunoscience (MI3), Medical Faculty Mannheim, Heidelberg University, Mannheim, Germany; 2 NGS Core Facility, Medical Faculty Mannheim, Heidelberg University, Mannheim, Germany

**Keywords:** Gene expression profiling, Natural killer cells, Pathway analysis, Sepsis, Systemic inflammatory response syndrome

## Abstract

**Background:**

Natural killer (NK) cells exert cytotoxicity against transformed and infected cells. In human sepsis, a suppressive NK cell receptor signature and defective effector molecule expression have been described. However, the transcriptional mechanisms underlying this phenotype remain poorly defined.

**Methods:**

We analyzed microarray-based transcriptomic profiles of isolated peripheral NK cells from patients with sepsis, patients with systemic inflammatory response syndrome (SIRS), and presurgical controls. Enrichment analyses of canonical pathways, biological processes, and cellular compartments were performed. Differential gene expression was validated in an independent cohort using a multiplex branched-DNA assay. Functional signal transducer and activator of transcription (STAT) phosphorylation responses ex vivo and proliferation marker expression were assessed by flow cytometry in independent patient samples.

**Results:**

NK cells from patients with sepsis displayed transcriptional signatures indicative of DNA replication stress, endoplasmic reticulum (ER) stress, altered cytoskeletal dynamics, and vesicle trafficking. Despite enrichment of proliferation-associated transcriptional programs, NK cells showed no increase in Ki-67 expression, indicating impaired proliferative activity. In contrast, NK cells from patients with SIRS exhibited downregulation of immune signaling pathways.

**Conclusion:**

This study identifies early stress-associated transcriptional programs and impaired subcellular organization in circulating NK cells during sepsis. Dysregulated DNA replication and ER stress responses, along with altered vesicle trafficking linked to impaired small guanosine triphosphatase (GTPase) signaling, may contribute to NK cell dysfunction in sepsis and may inform the development of NK cell-based immunotherapeutic strategies in critical illness.

**Supplementary Information:**

The online version contains supplementary material available at 10.1186/s11658-025-00851-2.

## Introduction

Sepsis is a dysregulated host response to an infection that causes life-threatening organ dysfunction [1]. It has an estimated in-hospital mortality of 27% [2]. Timely source control, antimicrobial therapy, and hemodynamic stabilization are life-saving [3]. Despite improvements in acute and supportive care [4, 5], hyperinflammation in sepsis is rapidly followed by systemic immunosuppression [6, 7]. Failure to regain immune homeostasis is associated with prolonged intensive care and enhanced susceptibility to secondary infections [8, 9].

Natural killer (NK) cells exert natural cytotoxicity against infected and transformed cells and promote adaptive immunity by releasing interferon γ (IFN-γ) and other cytokines [10]. During maturation in the bone marrow and secondary lymphoid organs, NK cells become committed to expressing heterogeneous combinatorial repertoires of germline-encoded stimulatory and inhibitory receptors to recognize and kill stressed while sparing healthy cells [11].

Limited information is available on NK cells in sepsis [12]. A very early increase in their blood counts and elevated surface levels of the activation marker CD69 have been reported to correlate with increased mortality [13–15]. However, already within the first day after onset, sepsis-induced lymphopenia is marked by reduced NK cell counts, likely attributable to regulated cell death (RCD) and tissue redistribution [15, 16]. Peripheral NK cells show impaired cytolytic NK effector molecule expression and ex vivo IFN-γ production. Souza-Fonseca-Guimaraes et al. (2012) found this to hold for patients admitted to the intensive care unit (ICU) with sepsis and systemic inflammatory response syndrome (SIRS) [17]. On ICU days 3–5 following sepsis onset, both CD56^dim^ and CD56^bright^ NK cells showed proportionate reductions in the blood lymphocytes of patients with sepsis compared with healthy donors [18]. At the same time, the activated phenotype reflected by elevated CD69 and additionally NKp44 persisted. Ex vivo production of IFN-γ and tumor necrosis factor-α (TNF-α) was reduced in both NK cell subpopulations. Furthermore, the proportions of CD56^dim^ and CD56^bright^ NK cells positive for the stimulatory NKG2 receptor family members C and D and binding to an antibody recognizing killer immunoglobulin-like receptor family members KIR2DL1/S1/S3/S5 were reduced. In contrast, the proportion of inhibitory NKG2A-positive CD56^dim^ NK cells was elevated [18]. In patients with sepsis on ICU admission, an increase in the percentage of programmed cell death ligand 1-positive NK cells predicted 28-day mortality [19]. Together, these reports support a suppressed NK cell phenotype in sepsis.

Notably, there is a shortage of transcriptional correlates for this phenotype, likely owing to challenges in accessing NK cells during sepsis-induced lymphopenia. Reported single-cell RNA-sequencing (scRNA-seq) analyses performed on peripheral blood mononuclear cells (PBMCs) from ICU patients with sepsis were focused on myeloid cells [20–26] or T cells [27, 28]. For NK cells, features of exhaustion and apoptosis were described from such analyses in patients with sepsis with Gram-negative pneumonia [29], and reduced levels of several NKG2 receptor genes in late sepsis after surgery or trauma [28], each compared with healthy donors. An analysis of scRNA-seq data from PBMCs after severe violent trauma attributed reduced NK cell blood counts predominantly to apoptosis, pyroptosis, and other forms of RCD [30]. However, scRNA-seq approaches relying on whole blood or PBMCs provide high-resolution insights only for abundant populations or aggregate transcriptomic signals from different cell types while missing signals from minor but important populations such as NK cells.

In the current transcriptome study, we thus resorted to our reported microarray dataset of magnetically separated NK cells from the blood of patients with sepsis and SIRS on admission to a surgical ICU (discovery cohort) [31]. While most of the reported analyses on NK cells in sepsis included healthy controls [14, 16, 18, 28, 30], we enrolled patients at their presurgical examination as controls. We conducted enrichment analyses of canonical pathways, biological processes, and cellular compartments, and identified differentially expressed genes (DEGs). To validate our findings, differential gene expression was confirmed by a multiplex branched DNA assay in NK cells from an independent cohort of patients with sepsis and SIRS on ICU admission (validation cohort). Gene clustering was followed by correlation analyses of gene expression and clinical features of organ dysfunction and inflammation. In a third cohort, including sepsis, SIRS, and healthy donors, we assessed functional immune signaling and expression of the proliferation marker Ki-67 by flow cytometry. The transcriptional signature of NK cells indicated an activated state in sepsis and immune signaling suppression in SIRS. Our results point toward replication stress, ER stress, and impaired subcellular organization due to altered regulation by small GTPases as potential contributors to NK cell suppression in sepsis. These pathomechanistic insights could inform NK cell-based therapeutic strategies in critical illness.

## Materials and methods

### Study participants

The study was approved by the Medical Ethics Commission II of the Medical Faculty Mannheim, Heidelberg University, Germany (2011-411M-MA; 2016-521N-MA; 2020-587N) in accordance with the Helsinki Declaration. All participants or their legal representatives provided written informed consent.

The patients were from three monocenter prospective cohorts recruited on admission to the anesthesiology-led interdisciplinary surgical adult ICU of the University Medical Center Mannheim (UMM) [31, 32]. The cohorts included patients with posttraumatic SIRS and sepsis according to Sepsis-1/2 [33], who concurrently fulfilled the Sepsis-3 definition [1]. The first, referred to as the discovery cohort, additionally included presurgical controls. It contributed the NK cell microarray dataset [31] for bioinformatics analysis. The second (validation) cohort provided NK cell lysates stored at −80 °C [31] for confirmation of differential gene expression. Blood samples from the third cohort [32] and additionally from healthy donors were subjected to flow cytometric phosphoprotein (pSTAT3/pSTAT5) and proliferation marker (Ki-67) determinations.

### NK cell isolation

NK cells isolated from the blood of patients admitted to the ICU with acute critical illness using conventional one-step magnetic bead-based isolation protocols show significant contaminations with granulocytes and monocytes due to marked emergency myelopoiesis. Therefore, we isolated NK cells from the discovery and validation cohort patients by a two-step MicroBead-based isolation protocol detailed recently [34]. In brief, 32 mL of EDTA-anticoagulated blood were collected within 24 h of ICU admission. The PBMCs were initially depleted of non-NK cells, followed by enrichment of NK cells using CD56 MicroBeads (Miltenyi Biotec, Germany), yielding on average 96.2% pure NK cells with less than 1% granulocytes, monocytes, T cells, and B cells each in flow cytometric analysis. The purified cells were counted and their viabilities were determined by trypan blue dye exclusion to be 94.3% on average using a Countess™ II automated cell counter (Invitrogen, Thermo Fisher). The same procedure was applied with the presurgical patients.

### Gene expression analyses

#### Bioinformatics analysis

We analyzed Gene Expression Omnibus database entry GSE123730 with 24,733 probes. Gene set enrichment analysis (GSEA) [35] was performed with the Kyoto Encyclopedia of Genes and Genomes (KEGG) and Gene Ontology (GO) databases by using the “genekitr” package [36] considering only pathways that can be attributed to NK cells. Differential expression was assessed using one-way analysis of variance (ANOVA) with the “limma” package [37].

All statistical procedures were conducted with the R programming language (R version 4.4.1) [38]. Gene set enrichment was based on a false-positive rate of < 0.05 with false discovery rate correction (FDR-*q* value) in pairwise patient group comparisons. We did not consider gene sets representing pathways and functions of specialized nonimmune cells. Thereby, we excluded, for instance, KEGG pathways from the functional class of “human diseases” and pathways such as “thermogenesis” or “oocyte meiosis.” For the analysis in GO, we assumed a normalized enrichment score (NES) of zero for enrichments with an FDR-*q* value ≥ 0.1. DEGs were also defined by an FDR-*q* value < 0.05 from pairwise patient group comparisons. To avoid redundancy, only a single GO term was considered if several terms featured the same core enrichment.

#### Confirmation of differential gene expression by QuantiGene Plex (QGP) assay

NK cell lysates from our validation cohort were subjected to QGP analysis on a magnetic bead array platform (MAGPIX^®^, Luminex Corporation, USA) as described using *AKIRIN1* as reference gene [31]. The RefSeq IDs and functional gene annotations are given in Additional file 1: Table S1.

#### Resources for gene function

For the cursory descriptions of DEGs in the “Results” section, information on their functions was sourced from GeneCards (www.genecards.org) [39] and miRBase (www.mirbase.org) [40].

### Flow cytometry

Data were acquired on a FACSLyric flow cytometer (BD Biosciences, USA) and analyzed with FlowJo™ V10 (Tree Star, USA). Details on the antibody–fluorochrome conjugates used are given in Additional file 1: Table S2.

To assess the phosphorylation status of signal transducer and activator of transcription (STAT) 3 and STAT5, heparin-anticoagulated blood was analyzed using the BD Phosflow protocol for human whole blood (BD Biosciences). Briefly, 200 µL blood was (i) either left unstimulated, (ii) stimulated with interleukin 15 (IL-15) (PeproTech, USA) at 45 ng/mL, or (iii) stimulated with a combination of IL-15 at 45 ng/mL and IL-18 (MBL International (USA) at 50 ng/mL for 15 min at 37 °C (INCU-Line IL23 incubator, VWR, USA). CD3-FITC and CD56-PE were added simultaneously. Samples were fixed with 2 mL Phosflow Lyse/Fix Buffer for 11 min at 37 °C, washed with PBS (6 min, 600 × *g*), and permeabilized with 1 mL Perm Buffer III for 30 min on ice. The samples were washed twice with Stain Buffer (600 × g, 6 min) and either stained with Phosflow antibodies [BV421-STAT3 (pS727) and AF647-STAT5 (pY694)] or isotype controls for 30 min at room temperature (RT). Samples were washed with 4 mL Stain Buffer (6 min, 600 × g) before resuspension in 250 μL Stain Buffer and acquisition of 10^5^ events in the CD3^−^CD56^+^ gate.

To determine the proliferation marker Ki-67, 100 µL of EDTA-anticoagulated blood were stained with FITC-CD3, PE-CD56, and PerCP-CD45 for 15 min at RT, followed by the addition of 2 mL BD FACS Lysing Solution and subsequent incubation for 10 min at RT. Samples were washed twice (500 × *g*, 5 min) with 2 mL BD CellWASH before the addition of 250 µL BD Fixation/Permeabilization solution and subsequent incubation for 20 min at 4 °C. After two washes with 1 mL BD Perm/Wash Buffer (300 × g, 5 min, 4 °C), the cell pellet was resuspended in 100 µL BD Perm/Wash Buffer before adding Ki-67 BV510 or the isotype control (IgG1κ BV510). Samples were incubated for 30 min at 4 °C and washed twice with 1 mL BD Perm/Wash Buffer (300 × *g*, 5 min). Cells were then resuspended in 250 µL BD CellWASH; 10^5^ events for CD45-positive cells were acquired.

### Statistical analysis

Continuous and dichotomous variables for clinical characteristics were compared with the *t*-test and chi-squared test, respectively, using SAS version 9.4 (Statistical Analysis System, SAS Institute, Cary, NC, USA). The effects of in vitro cytokine treatments of blood on the phosphorylation of STATs in NK cells were assessed with the Friedman test and Dunn’s post hoc test. Patient subgroup differences in STAT phosphorylation and Ki-67 expression were assessed with the Mann–Whitney *U* test using Prism 7 (GraphPad Software, USA). Spearman’s correlation coefficients were calculated with SigmaPlot version 11.0 (Systat Software Inc., USA) and interpreted according to Schober et al. (2018) [41]. The Bonferroni correction for multiple testing was applied where indicated. *P* values < 0.05 were considered statistically significant.

## Results

### Patient characteristics

Patient demographics and clinical phenotypes were representative for Western European ICUs [42]. Detailed characteristics have been reported previously for both the discovery (sepsis, *n* = 10; SIRS, *n* = 16; presurgical, *n* = 19) and validation (sepsis, *n* = 15; SIRS, *n* = 18) cohorts [31]. Across subgroups, mean ages ranged from 62 to 72 years and male proportions from 47% to 75%. In patients included in flow cytometric analyses of STAT3 and STAT5 phosphorylation (Phosflow; sepsis, *n* = 19; SIRS, *n* = 11) and of the proliferation marker Ki-67 (sepsis, *n* = 33; SIRS, *n* = 18), mean ages ranged from 63 to 68 years, and male proportions from 45% to 61% (Additional file 1: Table S3). Healthy donors (*n* = 10) were younger (mean age 48 years, 70% male).

Lung and abdomen were the predominant sites of infection in patients with sepsis of the discovery and validation cohorts [31]. In flow cytometry cohorts, infection foci were more diverse, with bloodstream (32%) and abdomen (27%) being most frequent in Phosflow and Ki-67 subcohorts, respectively. Abdominal surgery accounted for SIRS in 63% of the discovery and 39% of the validation cohort patients, each followed by thoracic surgery [31]. Abdominal surgery remained the most frequent cause of SIRS in the Phosflow (73%) and Ki-67 (56%) subcohorts (Additional file 1: Table S3).

The mean sequential organ failure assessment (SOFA) scores on study inclusion were significantly higher in sepsis than SIRS, both in discovery (13.1 versus 5.3) and in validation (10.4 versus 6.3) cohorts [31]. Similarly, C-reactive protein (CRP) levels were elevated in patients with sepsis in both cohorts, while white blood cell (WBC) counts did not differ significantly. In the Phosflow subcohort, mean SOFA was 7.1 versus 3.7 (*p* = 0.019), and in the Ki-67 subcohort 6.4 versus 4.4 (*p* = 0.059) (Additional file 1: Table S3). Mean WBC counts were 1.5-fold (*p* = 0.062) higher in sepsis than SIRS in the Phosflow and 1.4-fold (*p* = 0.062) higher in the Ki-67 subcohorts. Mean CRP levels in sepsis were 1.9-fold (*p* = 0.003) and 2.4-fold (*p* < 0.001) higher than in SIRS, accordingly. Hence, the Phosflow and Ki-67 subcohorts differed from the discovery/validation cohorts mainly by lower SOFA means in patients with sepsis, while relative sepsis–SIRS differences were overall similar.

### Pathway enrichment reveals positive regulation of cell proliferation and DNA repair in sepsis and negative regulation of immune signaling in SIRS

**Fig. 1:**
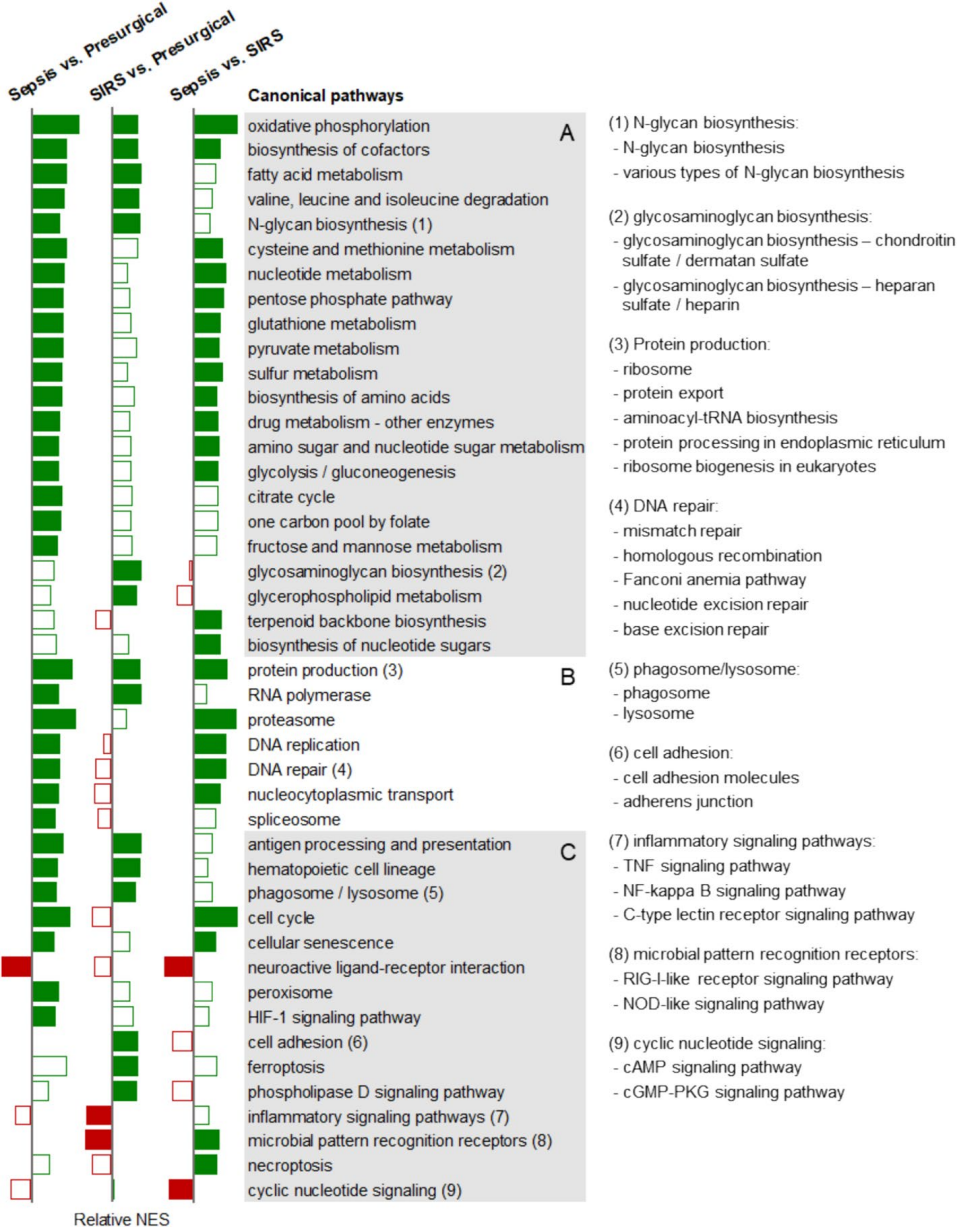
Canonical pathway enrichment identifies differential pathway regulation in NK cells from patients with sepsis and SIRS. Bar chart summarizing the relative normalized enrichment scores (NES) of cellular canonical pathways from the KEGG database in NK cells from patients with sepsis (*n* = 10) and SIRS (*n* = 16) on ICU admission and from presurgical controls (*n* = 19). Filled bars indicate a false discovery rate-*q* value < 0.05, green bars positive and red negative associations. Pathways are arranged by KEGG category (**A**: metabolism, **B**: genetic information processing, **C**: environmental information processing, cellular processes, and organismal systems). The consecutive numbers designate groups of related pathways with a similar association profile and identified to the right and in Additional file 1: Table S4. The group means of the NES values are plotted. The results are further detailed in Additional file 1: Figs. S1, S2, S3.

We compared the microarray-based transcriptomes of NK cells from patients with sepsis (*n* = 10), patients with SIRS (*n* = 16), and presurgical controls (*n* = 19) by gene set enrichment analysis. Focusing on canonical pathways from the KEGG database, we identified statistically significant pathway associations for both sepsis and SIRS relative to controls (Fig. 1; Additional file 1: Table S4).

Sepsis was positively associated with 48 significantly enriched pathways (median normalized enrichment score [NES]: 1.99), including oxidative phosphorylation and ribosome as the leading pathways (NES > 3) (Additional file 1: Fig. S1A, B). SIRS showed 24 enriched pathways (NES: 1.79), primarily involving ribosome (NES > 2) (Additional file 1: Fig. S2B). Seventeen pathways were enriched in both sepsis and SIRS, related to energy, branched amino acid and N-glycan metabolism, protein production, RNA polymerase, antigen presentation, hematopoiesis, and lysosome. Twenty-six additional pathways enriched in sepsis compared with controls were consistent with an enhancement of central energy metabolism, cell anabolism, post-transcriptional regulation, protein turnover, cell proliferation, DNA repair, and chemical and hypoxic stress responses, including enrichment of the hypoxia-inducible factor 1 (HIF-1) signaling pathway (Fig. 1; Additional file 1: Fig. S1). Additional SIRS-associated pathways included glycosaminoglycan biosynthesis, glycerophospholipid metabolism, cell adhesion molecules, adherens junction, ferroptosis, and phospholipase D signaling pathway. SIRS showed negative enrichment of inflammatory signaling pathways, including tumor necrosis factor (TNF), nuclear factor kappa B (NF-κB), and C-type lectin receptor signaling, and microbial pattern recognition receptors (PRR) (Fig. 1; Additional file 1: Fig. S2). In the comparison of sepsis versus SIRS, statistical significance was reached for positive associations of terpenoid backbone biosynthesis, biosynthesis of nucleotide sugars and necroptosis. Sepsis also showed positive associations with microbial PRR (e.g., retinoic acid-inducible gene-I (RIG-I)-like receptor and nucleotide-binding oligomerization domain (NOD)-like signaling), while negative associations were observed with cyclic adenosine monophosphate (cAMP)/cyclic guanosine monophosphate (cGMP) signaling and neuroactive ligand–receptor interaction pathways (Fig. 1; Additional file 1: Fig. S3).

### Differential regulation of cell cycle checkpoint signaling, cytoskeletal organization, and vesicle transport in sepsis and SIRS

**Fig. 2:**
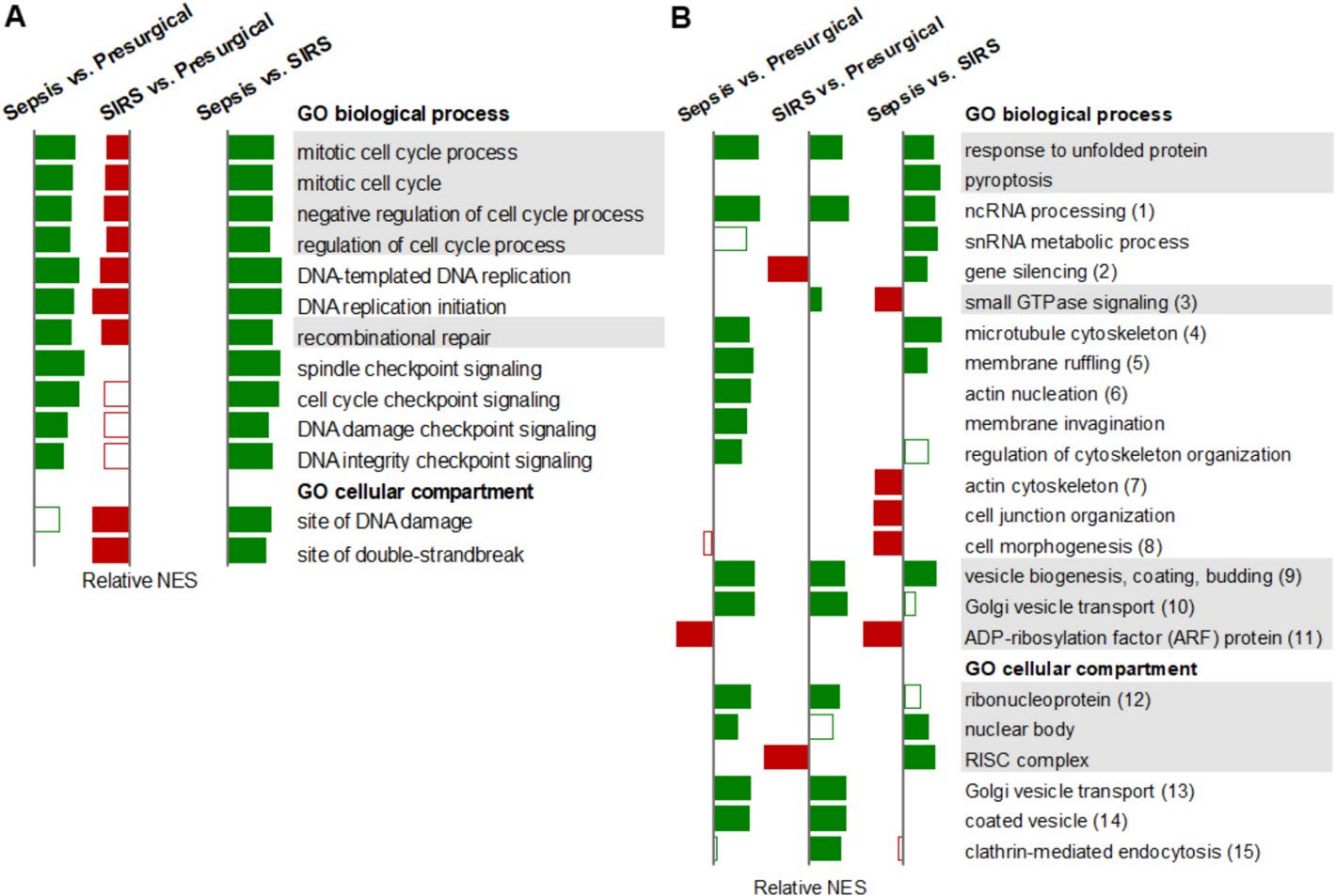
Gene Ontology (GO) term enrichment reveals differential regulation of cell cycle checkpoint signaling, cytoskeletal organization, and vesicle transport in sepsis and SIRS. The relative normalized enrichment scores (NES) for selected GO terms and groups of GO terms in NK cells from patients with sepsis (*n* = 10) and SIRS (*n* = 16) on ICU admission and from presurgical controls (*n* = 19) are shown as a bar chart. Filled bars indicate a false discovery rate-*q* values < 0.05, green bars positive and red negative associations. The grey background shading highlights related functions. **A** Cell cycle-, DNA replication-, DNA repair-, and checkpoint signaling-related functions. **B** Functions not represented by the canonical pathway analysis in Fig. 1. The consecutive numbers in parentheses designate groups of related functions with similar association profiles as identified in Additional file 1: Table S6 and Additional file 1: Figs. S4 and S5. Group means are plotted.

KEGG pathway analysis primarily aggregates common cellular functions into canonical pathways but provides limited cell type-specific activities. To gain more detailed insight into NK cell functional regulation, we performed GO term enrichment analysis for biological processes and cellular compartments. The results aligned with KEGG findings, yet the number of statistically significant associations of GO terms exceeded those of KEGG pathways by more than an order of magnitude. Both sepsis and SIRS, compared with presurgical controls, showed positive enrichment of GO terms related to protein production and central energy metabolism, including mitochondrial protein production/import and cell respiration. As with the KEGG pathways (Fig. 1), immune signaling was rather negatively associated with SIRS, extending particularly to type I IFN production and type II IFN signaling (Additional file 1: Table S5). Cell proliferation-, DNA repair-, and checkpoint signaling-related processes were positively associated with sepsis (Fig. 2A). Notably, except for cell division and several checkpoint signaling-related terms, these processes were negatively associated with SIRS, augmenting the contrast between sepsis and SIRS. The same applied to DNA damage-related cellular compartments.

GO terms not captured by KEGG analysis revealed additional enrichments (Fig. 2B; Additional file 1: Table S6; Figs. S4 and S5). Response to unfolded protein, noncoding RNA (ncRNA) metabolic process, and ncRNA processing were enriched in both sepsis and SIRS compared with controls and remained positively associated with sepsis when compared with SIRS.

Small nuclear RNA (snRNA) metabolic process and pyroptosis were positively associated specifically with sepsis compared with SIRS. While gene silencing processes and RNA-induced silencing complex (RISC) were enriched in sepsis compared with SIRS, they were negatively associated with SIRS.

Cytoskeleton-, cell shape-, and motility-related GO biological processes were differentially regulated in sepsis but unaffected in SIRS NK cells (Fig. 2B; Additional file 1: Table S6; Fig. S4). Microtubule (MT)-related pathways and membrane ruffling were enriched in sepsis compared with both controls and SIRS. Actin nucleation, membrane invagination, and regulation of cytoskeleton organization were enriched in sepsis versus controls only. Conversely, actin filament-based process, actin cytoskeleton organization, cell junction organization, and cell morphogenesis were negatively associated with sepsis versus SIRS. Small GTPase signaling-related processes exhibited negative associations in sepsis compared with SIRS (Fig. 2B; Additional file 1: Fig. S4C).

Vesicle trafficking pathways, including anterograde (COPII) and retrograde (COPI) transport between the endoplasmic reticulum (ER) and Golgi complex, were enriched in both sepsis and SIRS NK cells compared with controls (Fig. 2B; Additional file 1: Table S6; Figs. S4 and 5). In contrast, clathrin-mediated endocytosis and receptor recycling compartments were specifically enriched in SIRS. Additionally, adenosine diphosphate-ribosylation factor (ARF) protein-related pathways were negatively associated with sepsis compared with both SIRS and controls.

### Differential gene expression in sepsis and SIRS NK cells

**Fig. 3:**
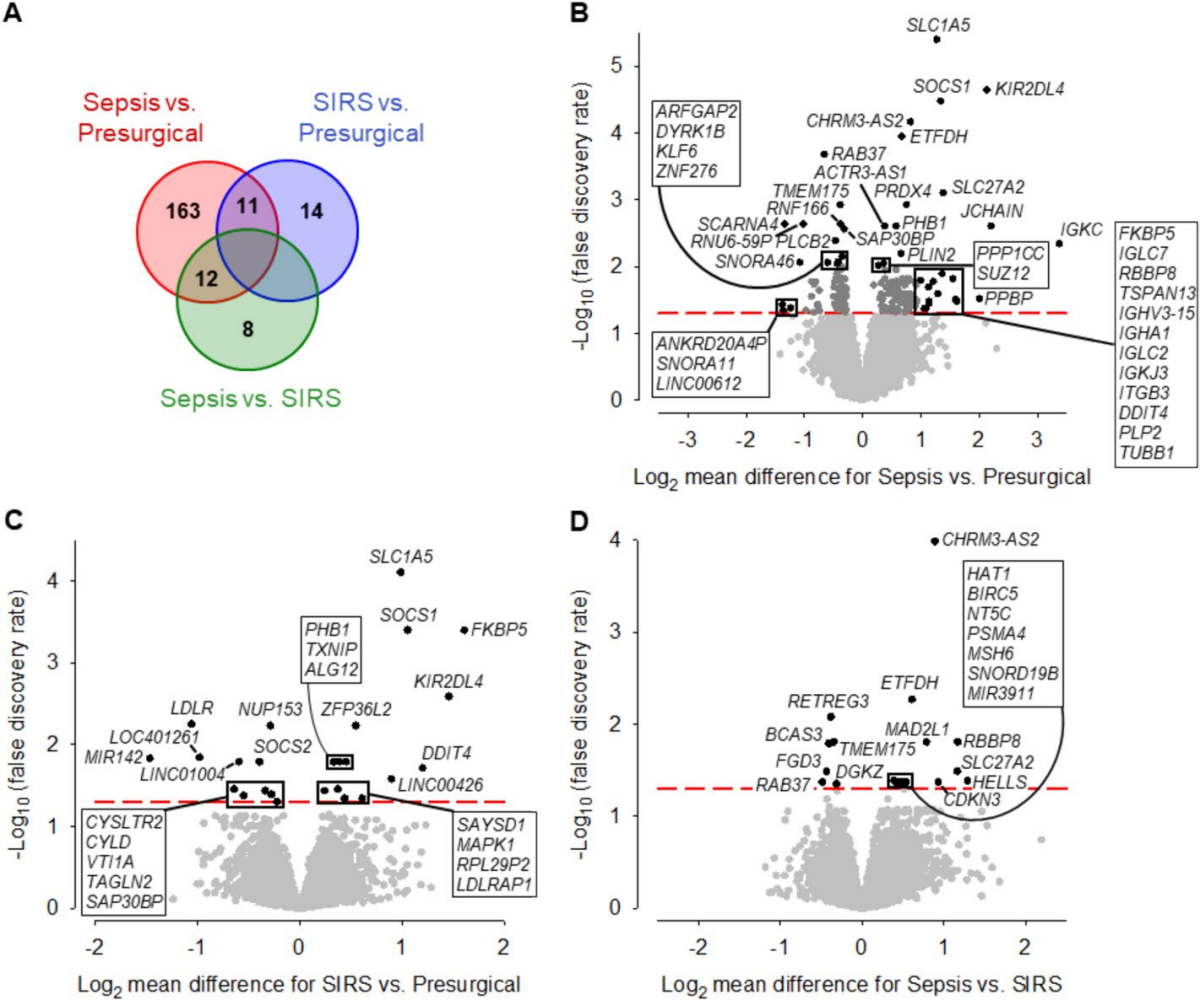
Differential gene expression in sepsis and SIRS NK cells. **A** Venn diagram for differentially expressed genes (DEGs) in the three pairwise patient subgroup comparisons: sepsis (*n* = 10), SIRS (*n* = 16), and presurgical controls (*n* = 19). Volcano plots display differential gene expression in (**B**) sepsis versus controls, **C** SIRS versus controls, **D** sepsis versus SIRS. In panel **B**, only DEGs with a mean difference > twofold and/or a false discovery rate (FDR) value < 0.01 are labeled. DEGs that stacked closely in the dot plots are surrounded by boxes and, therein, are listed by ascending FDR values. The dashed lines indicate the threshold for statistical significance (FDR < 0.05).

We identified a total of 208 DEGs across the three patient groups (Fig. 3A), with 186 DEGs distinguishing sepsis from presurgical controls. The 25 DEGs from the comparison of SIRS versus controls and the 20 DEGs from sepsis versus SIRS did not overlap. For the comparison of sepsis and controls, we focused on 42 DEGs showing over twofold mean expression difference and/or a *p*-value below 0.01 (Fig. 3B; Additional file 1: Fig. S6). The complete list of DEGs is available at 10.11588/DATA/IMEOVD.

Immune response genes elevated in both sepsis and SIRS included *SLC1A5*, *KIR2DL4*, *SOCS1*, and *FKBP5* (Fig. 3B, C). These genes are involved in NK cell activation and IFN-γ production, where *SLC1A5* encodes a glutamine transporter essential for NK cell effector functions [43] and *KIR2DL4* promotes a proinflammatory NK cell phenotype. *FKBP5* and *SOCS1* act in a negative feedback loop limiting IFN-γ production [44]. Additional sepsis-specific upregulation included *PPBP* (encoding CXCL7), *ITGB3* (integrin subunit β3), and ectopically expressed immunoglobulin genes (*IGKC*, *JCHAIN*, *IGKJ3*, *IGLC2*, *IGLC7*, *IGHA1*, and *IGHV3-15*) (Fig. 3B). In contrast, a negative NK signaling regulator, *DGKZ* [45], was expressed at lower levels in sepsis than SIRS (Fig. 3D). In SIRS, the levels of *CYLD* (negative regulator of NF-κB signaling in NK cells [46]), *SOCS2* (suppressor of NK cell differentiation [47]), and *CYSLTR2* were reduced compared with controls (Fig. 3C). Moreover, *PLCB2* and *KLF6* were expressed at lower levels in sepsis versus controls (Fig. 3B).

Sepsis NK cells also showed upregulation of metabolic (*SLC27A2* and *ETFDH*), protein turnover (*PSMA4*), and redox regulator (*PRDX4*) genes (Fig. 3B, D). Genes of chromatin regulation (*SAP30BP* and *SUZ12*) showed differential and small nucleolar RNAs (*SNORA11* and *SNORA46*) reduced levels in sepsis. *MIR3911* and *CHRM3-AS2* were elevated in sepsis, consistent with the transcriptomic hallmarks identified through GO term analysis. *CHRM3-AS2* was by far the most consistently elevated gene in sepsis compared with SIRS (Fig. 3D).

Genes controlling cell cycle progression and DNA repair elevated in sepsis compared with SIRS included *RBBP8*, *CDKN3*, *MAD2L1*, *BIRC5*, *HELLS*, *MSH6*, and *HAT1* (Fig. 3D). Vesicle trafficking and autophagy genes (*RAB37*, *ARFGAP2*, *TMEM175*, and *RNF166*) were downregulated in sepsis compared with controls (Fig. 3B). Lower levels in sepsis than SIRS were also detected for *RAB37* and *TMEM175* and additionally for *FGD3* and *BCAS3* (Fig. 3D). The most consistent decrease between sepsis and SIRS was observed for reticulophagy receptor gene *RETREG3* (Fig. 3D).

### NK cell gene signature distinguishes sepsis from SIRS

**Fig. 4:**
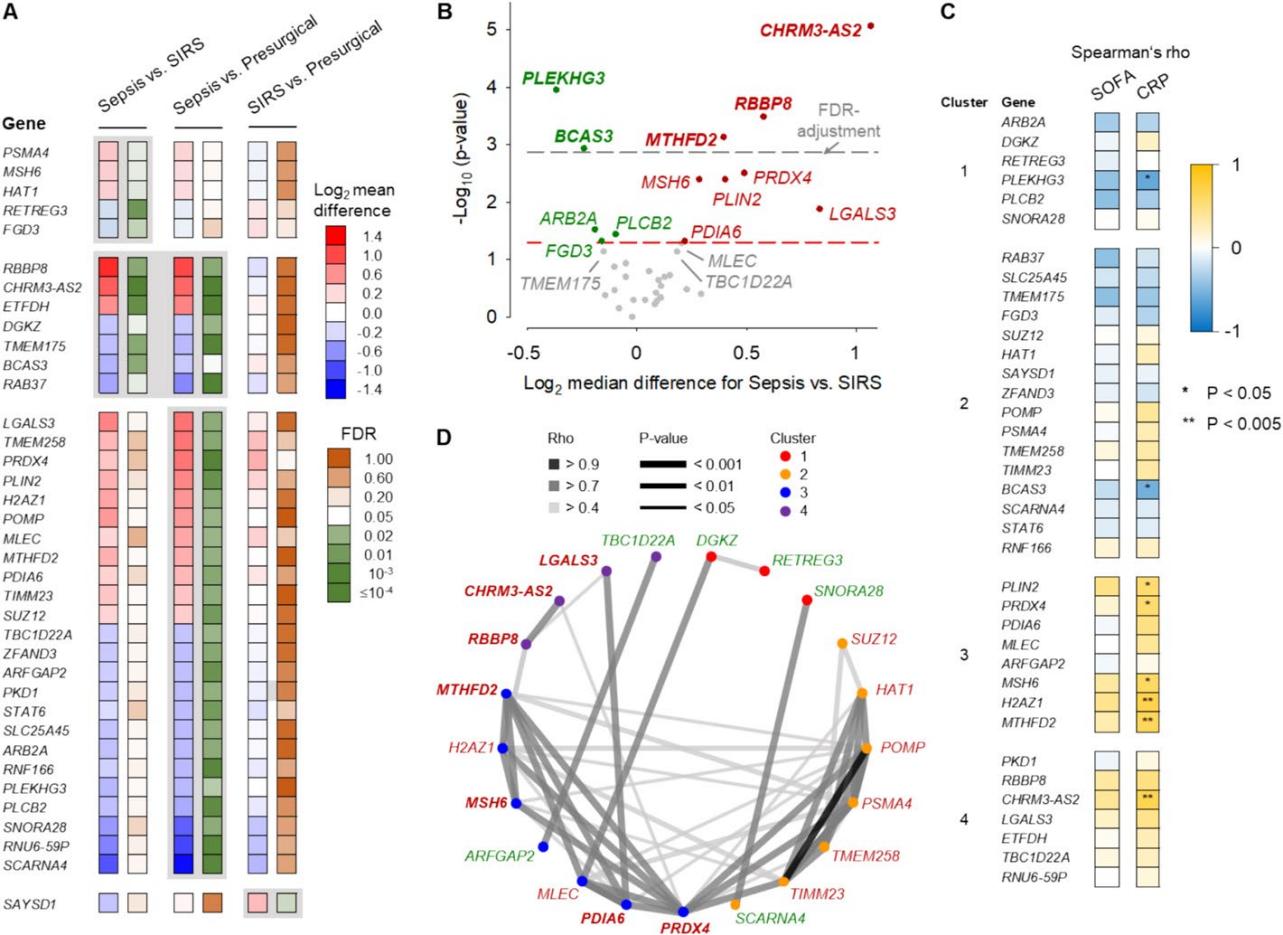
NK cell sepsis gene signature. **A** Heatmap of mean expression level differences and false discovery rate (FDR) values of differentially expressed genes selected to distinguish sepsis (*n* = 10) from SIRS (*n* = 16) and presurgical (*n* = 19) NK cells in the discovery cohort. Gray background shading highlights statistically significant differences. **B** Volcano plot of the QuantiGene Plex test results of the NK cell sepsis gene signature in the validation cohort patients with sepsis (*n* = 15) and SIRS (*n* = 18). The threshold for statistical significance (*p* = 0.05) in the unadjusted and Bonferroni-adjusted analysis is marked by the red and gray dashed line, respectively. Genes with higher and lower sepsis than SIRS levels are labeled in red and green, respectively. Three additional genes with differences at the borderline of statistical significance are labeled in gray. **C** The correlations between SOFA and CRP with the NK cell sepsis signature genes in the validation cohort (*n* = 33) are summarized as a heatmap representation of Spearman’s rho values. The genes are ordered according to the hierarchical clustering result (Additional file 1: Fig. S8). Asterisks indicate statistically significant correlations. **D** Genes with significant intergene correlations in the QuantiGene Plex analysis are arranged by cluster membership. Genes with higher and lower mean levels in sepsis than SIRS in the discovery cohort are printed in red and green, respectively, and in bold if the difference was confirmed in the validation cohort (panel **B**).

A signature of 37 genes from all 208 distinguished NK cells from patients with sepsis from those without sepsis in the discovery cohort by hierarchical clustering (Additional file 1: Fig. S7; Table S1). Across the signature, the sepsis–SIRS differences highly resembled the sepsis–control differences (Fig. 4A).

Validation via branched DNA-based signal amplification in an independent patient cohort revealed four main clusters (1–4) (Additional file 1: Fig. S8) and confirmed expression differences for 13 DEGs (Fig. 4B; Additional file 1: Fig. S9). Higher sepsis than SIRS levels were confirmed for *CHRM3-AS2*, *RBBP8*, *PRDX4*, *PLIN2*, and *MSH6*. As in the discovery cohort, *CHRM3-AS2* showed the most consistent upregulation in sepsis. Additional sepsis-upregulated genes included *MTHFD2*, *LGALS3*, and *PDIA6*. In contrast, downregulated genes included *BCAS3* and *FGD3*, encoding activators of CDC42, as well as *PLCB2*, encoding a calcium regulator activated by CDC42. The CDC42 activator gene *PLEKHG3* and *ARB2A*/*FAM172A* were downregulated in sepsis compared with SIRS.

Correlation analyses revealed a moderate positive correlation between SOFA and blood CRP (Spearman’s *ρ* = 0.672, *p* = 1.21 × 10^–5^) in the validation cohort (Additional file 1: Fig. S10A). Unsurprisingly, the correlations of the 37 NK cell sepsis signature genes with SOFA and with blood CRP showed overall similar profiles (Fig. 4C). Signature genes including *H2AZ1*, *CHRM3-AS2*, *MTHFD2*, *MSH6*, *PRDX4*, and *PLIN2* correlated positively with CRP; *PLEKHG3* and *BCAS3* showed negative correlations (Fig. 4C, Additional file 1: Fig. S10B). Considering sepsis and SIRS separately, only the CRP–*H2AZ1* correlation in SIRS reached statistical significance in unadjusted analyses (Additional file 1: Fig. S11).

Gene expression analysis revealed 48 significantly correlated gene pairs (7.21%), including strong intracluster correlations involving mitochondrial (*MTHFD2* and *TIMM23*), proteasomal (*POMP* and *PSMA4*), DNA repair-related (*HAT1*, *MSH6*), and redox-regulatory genes (*PDIA6* and *PRDX4*) (Fig. 4D; Additional file 1: Fig. S12). *PRDX4* stood out as a central hub, linking stress response modules across clusters 2 and 3. *CHRM3-AS2*, the gene with the most consistent elevation in sepsis compared with SIRS NK cells in both the discovery (Fig. 3D) and the validation cohort (Fig. 4B), showed a strong correlation with *RBBP8* within cluster 4. The most strongly correlated pair was *POMP*–*TIMM23*. Gene pairs with confirmed differential expression and strong correlation in the validation cohort (Fig. 4D) showed significant correlations in both patient subgroups in the unadjusted analyses. The only exceptions in the sepsis group were the pairs *RBBP8*–*CHRM3-AS2* and *PDIA6*–*LGALS3*, which did not reach significance (Additional file 1: Fig. S13).

### Elevated STAT3 baseline phosphorylation and low proliferation in sepsis NK cells

**Fig. 5:**
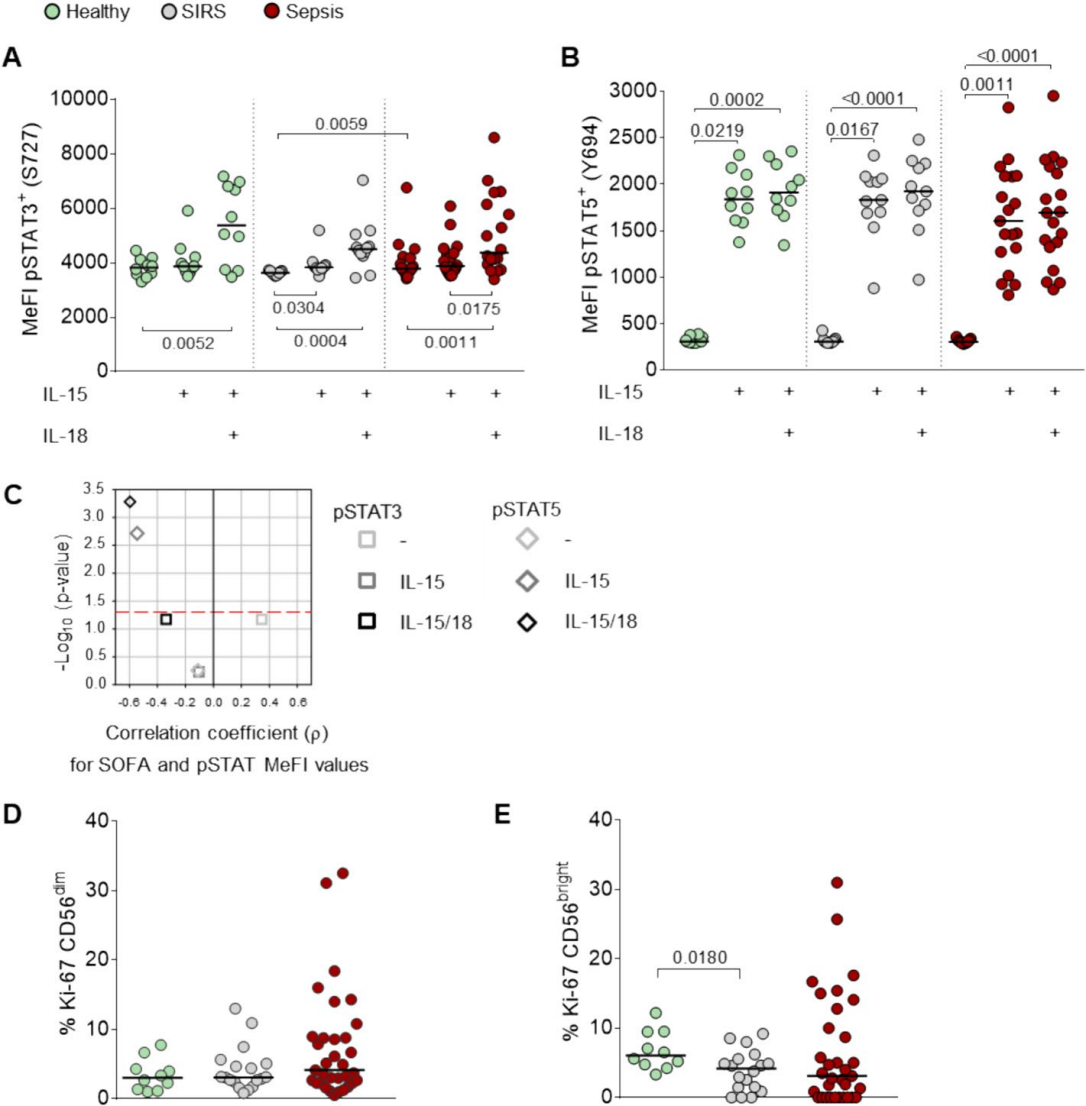
Sepsis NK cells show slightly increased STAT3 phosphorylation and a reduction in the median proportions of Ki-67-positive CD56^bright^ NK cells. The median fluorescence intensities (MeFIs) for pSTAT3 (S727) (**A**) and pSTAT5 (Y694) (**B**) were determined in whole blood by Phosflow and gating on CD3^−^CD56^+^ NK cells. The black horizontal lines indicate the median values. Blood from healthy donors (*n* = 10), patients with sepsis (*n* = 19), and patients with SIRS (*n* = 11) was treated for 15 min with cytokines as indicated. The shown *p*-values for the treatment effects are from the Friedman test with Dunn’s test and for patient subgroup differences from the Mann–Whitney *U* test. **C** Spearman’s correlation analysis results for the sequential organ failure assessment score (SOFA) and the pSTAT3 and pSTAT5 levels in NK cells from the patients (sepsis and SIRS combined). The negative common logarithm of the *p*-value is plotted against the correlation coefficient (*ρ*). The threshold for statistical significance (*p* = 0.05) is indicated by the dashed red line. The proportions of CD56^dim^ (**D**) and CD56^bright^ (**E**) NK cells positive for Ki-67 were determined by flow cytometry in whole blood from healthy donors (*n* = 10) and patients with sepsis (*n* = 33) and SIRS (*n* = 18). A significant difference is indicated by the *p*-value from the Mann–Whitney *U* test.

IL-15 activates NK cells via STAT3 and promotes their maturation, survival, and proliferation through STAT5 signaling [48], with synergistic enhancement by IL-18 [49, 50]. Using phospho-specific flow cytometry, we assessed baseline and IL-15/IL-18-induced phosphorylation of STAT3 (S727) and STAT5 (Y694) in NK cells from healthy donors and patients. Whole blood was stimulated with IL-15 alone or in combination with IL-18. Cytokine stimulation increased pSTAT3 and pSTAT5 positivity in all groups (Additional file 1: Fig. S14). STAT5 phosphorylation reached close to 100% across groups already with IL-15 alone, whereas STAT3 responses showed higher variability. Compared with untreated blood, the relative increase in pSTAT3 levels was less pronounced than in pSTAT5 (Fig. 5A, B). Notably, baseline STAT3 phosphorylation was slightly but significantly elevated in sepsis compared with SIRS (Fig. 5A). Cytokine stimulation increased the variance especially for pSTAT5 levels in sepsis (Fig. 5B).

Moreover, pSTAT5 responsiveness showed a moderate negative correlation with SOFA scores, suggesting that cytokine-induced STAT5 phosphorylation is impaired with increasing disease severity (Fig. 5C; Additional file 1: Fig. S15). Together, these data suggest that sepsis is associated with slightly higher baseline STAT3 phosphorylation than SIRS and that disease severity coincides with impaired STAT5 activation upon cytokine stimulation.

Despite the enrichment of proliferation-associated pathways (Fig. 1) and functions (Fig. 2A) in sepsis, protein-level analysis of the proliferation marker Ki-67 revealed low NK cell proliferative activity across all groups. Less than 10% of NK cells in both CD56^dim^ and CD56^bright^ subsets were Ki-67^+^ (Fig. 5D, E; Additional file 1: Fig. S16). Sepsis samples showed an increased variance, but median Ki-67 levels declined progressively from healthy controls to SIRS and sepsis, reaching statistical significance for CD56^bright^ NK cells of patients with SIRS (Fig. 5E). This suggests limited active proliferation of NK cells during sepsis.

## Discussion

In acute sepsis, NK cells exhibit reduced blood counts, a suppressive NK receptor signature, and defective effector molecule expression [15–19]. The transcriptional programs underlying this dysfunction remain poorly defined. To address this, we performed transcriptomic and targeted gene expression analyses on magnetically purified NK cells [34], rather than on whole blood or PBMCs as in previous studies [28–30]. NK cells were isolated from patients with postsurgical SIRS and from patients with a clinical diagnosis of sepsis within 24 h of ICU admission [31], also fulfilling the Sepsis-3 criteria [1]. Presurgical patients served as controls in the discovery set, providing a clinically more appropriate reference than healthy donors.

Transcriptional changes in immune pathways and genes in blood NK cells described here during early sepsis did not correspond to the known defects in NK cell effector molecule expression but rather indicated an activated cell state. Negative associations of cyclic nucleotide signaling (cAMP and cGMP-PKG) with sepsis suggest a disinhibition of NK cells [51, 52]. Moreover, the enrichment of the HIF-1 signaling pathway, known to support acute antimicrobial defense [53], in sepsis compared with controls (Fig. 1) is consistent with NK cell activation. The expression levels of several DEGs associated with NK cell activation were elevated compared with controls: in both sepsis and SIRS (*SLC1A5*, *KIR2DL4*, *SOCS1*, and *FKBP5*), in sepsis (*PPBP* and *ITGB3*), and in SIRS (*MAPK1*) (Fig. 3B, 3 C). Further supporting NK cell activation, decreased expression of negative regulators was observed in sepsis (*DGKZ*) (Fig. 3D) and in SIRS (*CYLD* and *SOCS2*) (Fig. 3C), suggesting NK cell disinhibition. Nevertheless, the increased expression of the Polycomb group protein coding gene *SUZ12* in sepsis (Fig. 3B) may enhance H3K27 methylation, which reportedly represses the production of IFN-γ, TNFα, granulocyte–macrophage colony-stimulating factor (GM-CSF), and IL-10 in NK cells [54].

In contrast, the downregulation of PRR signaling and central inflammatory signaling (TNF and NF-κB) in SIRS (Fig. 1) suggests NK cell inhibition. Elevated *ZFP36L2* expression in SIRS compared with controls (Fig. 3C) may limit cytokine production through the cytosolic decay of IFN-γ, TNFα, GM-CSF, and IL-3 mRNAs [55, 56]. Additionally, lower *TAGLN2* levels in SIRS may impair NK cell cytotoxicity as reported for CD8 T cells [57]. Overall, these transcriptional profiles point toward an activated NK cell state in sepsis, which is also in line with an elevated baseline phosphorylation of STAT3 (Fig. 5A) and rather an inhibited state in SIRS.

Sepsis was associated with transcriptional signatures of unresolved cellular stress, including ER stress, replication stress, and nucleolar stress. Elevated expression of *PRDX4* and the ER-resident, protein coding genes *PLP2* and *PDIA6* (Fig. 3B, 4A, B) together with the enrichment of glutathione metabolism (Fig. 1) and response to unfolded protein (Fig. 2B), suggest compensatory responses to oxidative and ER stress. PRDX4 maintains redox balance in the ER [58]. In sepsis, circulating PRDX4 has been shown to correlate with both CRP levels and disease severity [59]. Consistent with this, *PRDX4* expression in NK cells correlated with CRP in our validation cohort (Fig. 4C). Moreover, elevated expression of the ER-redox-sensor and chaperone *PDIA6* (Fig. 4A, B) is consistent with the unfolded protein response (UPR) [60]. The UPR and ER stress also trigger reticulophagy where ER-anchored RETREG3 limits ER flux [61]. Sepsis-associated NK cells showed lower *RETREG3* expression compared with SIRS (Fig. 3D). This downregulation may impair the capacity of NK cells to resolve ER stress increasing their susceptibility to pyroptosis [62, 63], an RCD process positively associated with sepsis compared with SIRS (Fig. 2B). Additionally, necroptosis was associated with sepsis compared with SIRS, and ferroptosis with SIRS compared with controls (Fig. 1), both of which can be promoted by oxidative and mitochondrial stress. These findings point to distinct forms of regulated cell death contributing to peripheral NK cell loss in critical illness.

Blood lymphocytes in sepsis have been reported to feature an increased burden of DNA damage [64]. In line with this, we observed enrichment of multiple DNA repair, replication, cell cycle, and checkpoint signaling pathways (Fig. 1; Additional file 1: Figs. S1, S3; Table S4) and associated cellular compartments (Fig. 2). These were accompanied by elevated expression of genes for DSB repair (*RBBP8*, *PPP1CC*, *HELLS*, *HAT1*, and *H2AZ1*), mismatch repair (*MSH6*) (Figs. 3B, D, 4A, B), and cell cycle checkpoint genes (*CDKN3*, *MAD2L1*, and *BIRC5*) (Fig. 3D). None of these changes were seen in SIRS. At the same time, we did not observe elevated Ki-67 levels in patients’ NK cells (Fig. 5D, E). Instead, Ki-67 expression was low across all groups, with a trend toward reduced proportions of Ki-67^+^ CD56^bright^ NK cells in SIRS and sepsis (Fig. 5E). This agrees with limited proliferative activity and potential cell cycle arrest due to replication stress, to which NK cells are known to be particularly susceptible [65].

In parallel with responses to unresolved stress, we found elevated expression of genes involved in chromatin remodeling, and metabolic adaptation, consistent with early stress responses in sepsis-associated NK cells. Distinct alterations in RNA processing and gene silencing mechanisms were observed. Among these, *CHRM3-AS2*, whose function remains largely unexplored, was the most consistently upregulated gene in sepsis (Fig. 3D, 4B). By targeting miR-370-5p/KLF4, *CHRM3-AS2* was proposed to act as a glioma-associated oncogene [66]. Its co-expression with proliferation- and DNA repair-related transcripts such as *RBBP8* in sepsis supports its potential role in modulating NK cell state. The altered expression of small nucleolar (snoRNAs) and small nuclear RNAs (snRNAs) further suggests disruption of ribosomal RNA modification and spliceosome function, which may contribute to nucleolar stress and p53 activation [67].

The NK cell transcriptomes in sepsis and SIRS furthermore differed in the control of two subcellular processes by members of the Ras superfamily of small GTPases: cell migration and vesicle trafficking. Multiple genes functionally linked to CDC42 signaling, namely *FGD3* [68, 69], *PLEKHG3* [70, 71], *BCAS3* [72], and *PLCB2* [73], were downregulated in sepsis (Fig. 4B), suggesting impaired control of cell migration, polarity, and immune synapse formation. Additionally, the reduced expression of *ARFGAP2* (Fig. 3B) and negative association of the ARF protein pathway with sepsis (Fig. 2B) point to defective Golgi-to-ER trafficking [74]. These alterations in subcellular organization, in line with the reduced *RETREG3* expression (Fig. 3D) described above, may act in concert to compromise NK cell cytotoxicity and responsiveness during sepsis.

At the level of immune signaling, baseline phosphorylation of STAT3 (S727) was modestly elevated in sepsis NK cells (Fig. 5A). Together with the increase in circulating IL-6 [75], this finding is consistent with the potential impairment of NK cell function in sepsis via the IL-6−STAT3 axis [76]. STAT5 phosphorylation reached near-saturation in all groups upon cytokine exposure (Additional file 1: Fig. S14B) but showed greater variability in sepsis (Fig. 5B) and a negative correlation with SOFA scores (Fig. 5C). Baseline levels of pSTAT5 did not differ significantly between groups, though a trend toward lower phosphorylation from healthy donors to patients with SIRS and sepsis was observed. Elevated blood levels of IL-15 have been reported in sepsis [77]. Although the cytokine responsiveness of STAT3 and STAT5 appears preserved, STAT5 signaling becomes increasingly impaired with disease severity, possibly reflecting receptor desensitization, upregulation of inhibitory SOCS proteins [78], or exhaustion-like phenotypes. In addition to STAT3 and STAT5, IL-15 induces mTOR signaling in support of energy metabolism during NK cell activation [79]. In our KEGG pathway analysis, mTOR signaling was not significantly enriched in sepsis versus SIRS NK cells (*p* = 0.158). Nevertheless, the upregulation of energy metabolism- and stress-related genes, including enrichment of the HIF-1 signaling pathway, in sepsis does not exclude functional engagement of mTOR-associated metabolic programs.

The capacity for STAT3 and STAT5 activation provides a mechanistic rationale to investigate cytokine-based approaches aimed at improving NK cell stress resistance and survival for example through IL-15 [80]. However, potential risks such as systemic immune activation and a narrow therapeutic window must be considered. Together, our findings indicate that NK cell metabolism and redox balance represent potential targets for therapeutic modulation, which could be explored alongside immunomodulatory strategies such as checkpoint blockade (e.g., programmed death-1 (PD-1) or NKG2A inhibition) as implemented in antitumor NK cell therapies [81].

As a limitation, our bulk transcriptomic approach does not reveal potential changes in NK cell subpopulation composition and their contributions to the sepsis–SIRS differences described here. Future scRNA-seq will enable such analyses, for example, across classical CD56^dim^ and CD56^bright^ NK cells [82] or the more recently described NK1−NK3 subsets [83]. Given sepsis-induced lymphopenia, efficient NK cell enrichment will likely remain essential for scRNA-seq profiling. By focusing on enriched NK cells, our study enabled a detailed analysis of NK cell-intrinsic transcriptional alterations that are masked in PBMC or whole blood transcriptomes. Nevertheless, we cannot exclude that some of the identified pathway alterations are shared across several immune cell types. It further remains to be determined to what extent the reported transcriptional differences translate into functional changes. Flow cytometry can be used to assess immune signaling, stress responses and effector functions, including cytotoxicity and cytokine production, within NK cell subpopulations. These data can then be integrated with scRNA-seq profiles from corresponding NK cell subsets. Moreover, NK cell transcriptional and functional states may change rapidly in early sepsis. Longitudinal analyses will be required to capture the dynamic evolution of NK cell responses, which remains challenging owing to the persistent uncertainty in the timeliness of the sepsis diagnosis [84].

## Conclusions

This study identifies divergent transcriptional programs in NK cells during early sepsis and SIRS. Central immune signaling pathways were negatively associated with SIRS but not with sepsis, where an immune-activated state prevailed. The correlation of signature genes with clinical inflammation markers (e.g., CRP) supports their disease relevance. Our data identify the activation of responses to replication stress, ER stress, and nucleolar stress as potential contributors to several RCD programs in sepsis NK cells. In addition, disruption of small GTPase-controlled subcellular organization emerges as a novel vulnerability in sepsis that warrants further investigation.

## Supplementary Information

Additional file 1

## Data Availability

The results from the gene set enrichment, differential gene expression, multiplex, and flow cytometry analyses are available from 10.11588/DATA/IMEOVD.

## Abbreviations

CRP: C-reactive protein
DEG: Differentially expressed genes
EDTA: Ethylenediaminetetraacetic acid
ER: Endoplasmic reticulum
FDR: False discovery rate
GO: Gene ontology
GSEA: Gene set enrichment analysis
ICU: Intensive care unit
IFN-γ: Interferon-γ
IL: Interleukin
KEGG: Kyoto Encyclopedia of Genes and Genomes
NES: Normalized enrichment score
NK cells: Natural killer cells
NKG2: Natural killer group 2
mTOR: Mechanistic target of rapamycin
PBMC: Peripheral blood mononuclear cells
PRR: Pattern recognition receptor
QGP: QuantiGene Plex
RCD: Regulated cell death
RT: Room temperature
SIRS: Systemic inflammatory response syndrome
scRNA-seq: Single cell RNA sequencing
SOFA: Sequential organ failure assessment
STAT: Signal transducer and activator of transcription
TNF-α: Tumor necrosis factor α
UPR: Unfolded protein response
WBC: White blood cell
