# Supplementary material for: Sepsis alters NK cell transcriptional programs for stress, actin remodeling, and intracellular trafficking
Source: Cell Mol Biol Lett. 2026 Feb 4;31:29. doi: 10.1186/s11658-025-00851-2 (PMC12954936; doi:10.1186/s11658-025-00851-2)
Supplement: Supplementary file 1 — Additional file 1 [file 11658_2025_851_MOESM1_ESM.docx]

Supplementary Information

**Supplementary Table 1** GenBank Nucleotide IDs for QuantiGene^TM^ Plex Assays (Thermo Fisher Scientific) and functional gene annotations.

**Supplementary Table 2** Fluorochrome-conjugated monoclonal antibodies (BD Biosciences) used in flow cytometry.

**Supplementary Table 3** Demographics and clinical characteristics of the patients in the Phosflow and Ki-67 subcohorts.

**Supplementary Table 4** KEGG pathway enrichment analysis in NK cells from patients with sepsis, SIRS, and presurgical controls (GSE123730).

**Supplementary Table 5** Enrichment analysis of immune signaling related Gene Ontology (GO) biological processes in NK cells from patients with sepsis, SIRS, and presurgical controls (GSE123730).

**Supplementary Table 6** Enrichment analysis of additional Gene Ontology (GO) terms in NK cells from patients with sepsis, SIRS, and presurgical controls (GSE123730).

**Supplementary Figure 1** KEGG pathway enrichment analysis in NK cells from patients with sepsis and presurgical controls (GSE123730).

**Supplementary Figure 2** KEGG pathway enrichment analysis in NK cells from patients with SIRS compared to presurgical controls (GSE123730).

**Supplementary Figure 3** KEGG pathway enrichment analysis in NK cells from patients with sepsis compared to SIRS (GSE123730).

**Supplementary Figure 4** Enrichment analysis of Gene Ontology (GO) biological processes in NK cells from patients with sepsis, SIRS, and presurgical controls (GSE123730).

**Supplementary Figure 5** Enrichment analysis of Gene Ontology (GO) cellular compartments in NK cells from patients with sepsis, SIRS, and presurgical controls (GSE123730).

**Supplementary Figure 6** Differentially expressed genes in NK cells from patients with sepsis and presurgical controls with a mean difference ≤ twofold and a false discovery rate (FDR) between 0.01 and 0.5 (GSE123730).

**Supplementary Figure 7** Heatmap of microarray results (GSE123730).

**Supplementary Figure 8** Heatmap of QuantiGene Plex results.

**Supplementary Figure 9** NK cell sepsis signature genes with significant sepsis-SIRS differences in the validation cohort.

**Supplementary Figure 10** Correlations for blood C-reactive protein (CRP) in the validation cohort.

**Supplementary Figure 11** Correlations between blood C-reactive protein (CRP) and NK cell sepsis signature genes in the validation cohort.

**Supplementary Figure 12** Intergene correlations for the NK cell sepsis gene signature in the validation cohort.

**Supplementary Figure 13** Strong intergene correlations for genes with confirmed expression level differences in sepsis and SIRS NK cells in the validation cohort.

**Supplementary Figure 14** Quantification and representative flow cytometry analysis of STAT3 and STAT5 phosphorylation in NK cells from healthy donors and patients with sepsis or SIRS.

**Supplementary Figure 15** Correlations between SOFA score and pSTAT5 median fluorescence intensity (MeFI) values in sepsis and SIRS NK cells after cytokine treatment.

**Supplementary Figure 16** Representative flow cytometry analysis of Ki-67 in NK cell subsets.

**Supplementary Table 1** GenBank Nucleotide IDs for QuantiGene^TM^ Plex Assays (Thermo Fisher Scientific) and functional gene annotations.

| **Gene symbol** | **RefSeq^1^** | **Gene-/protein-associated function^2^** |
| --- | --- | --- |
| *AKIRIN1* | NM_024595 | *Reference gene used for signal normalization* |
| *ARB2A/FAM172A* | NM_032042 | ARB2 cotranscriptional regulator A, mRNA splicing |
| *ARFGAP2* | NM_032389 | ADP ribosylation factor GTPase activating protein 2, COPI-related |
| *BCAS3* | NM_017679 | Microtubule associated cell migration factor |
| *CHRM3-AS2* | NR_103776 | LncRNA, downregulates miR-370-5p in glioma cells^3^ |
| *DGKZ* | NM_003646 | Diacylglycerol kinase ζ, negative regulator of NK cells |
| *ETFDH* | NM_004453 | Electron transfer flavoprotein dehydrogenase |
| *FGD3* | NM_033086 | Regulation of actin cytoskeleton (predicted) |
| *H2AZ1* | NM_002106 | Nucleosomal DNA unwrapping and accessibility^4^ |
| *HAT1* | NM_003642 | Histone acetyltransferase 1, cell cycle, DNA repair |
| *LGALS3* | NM_002306 | Galectin 3, pre-mRNA splicing, lysophagy^5^ |
| *MLEC* | NM_014730 | Malectin, targets misfolded glycoproteins to degradation^6^ |
| *MSH6* | NM_000179 | DNA mismatch repair protein Msh6 |
| *MTHFD2* | NM_006636 | Methylenetetrahydrofolate dehydrogenase 2 |
| *PDIA6* | NM_005742 | Protein disulfide isomerase, inhibits the UPR^7^ |
| *PKD1* | NM_000296 | PKD1 forms Ca2^+^ channel with PKD2, mechanosensor |
| *PLCB2* | NM_004573 | Phospholipase C β2, inhibits TAK1 in viral infection^8^ |
| *PLEKHG3* | NM_001308147 | RhoGEF for Rac1/Cdc42, directed cell migration^9^ |
| *PLIN2* | NM_001122 | Perilipin 2, coating of cytoplasmic lipid droplets |
| *POMP* | NM_015932 | Proteasome maturation protein |
| *PRDX4* | NM_006406 | Peroxiredoxin 4, antioxidant enzyme, NK-κB activation |
| *PSMA4* | NM_002789 | Core alpha subunit of the 20S proteasome |
| *RAB37* | NM_001006638 | Small GTPase, degranulation and autophagy^10^ |
| *RBBP8* | NM_002894 | RB/BRCA1 associated nuclease, double-strand break repair |
| *RETREG3* | NM_178126 | Reticulophagy regulator family member 3 |
| *RNF166* | NM_178841 | Ring finger protein 166, polyubiquitination, autophagy |
| *RNU6-59P* | NR_046933 | RNA, U6 small nuclear 59, pseudogene |
| *SAYSD1* | NM_018322 | Translocation-associated quality control |
| *SCARNA4* | NR_003005 | Small Cajal body-specific RNA 4, pseudouridylation |
| *SLC25A45* | NM_182556 | Acyl carnitine transmembrane transporter (predicted) |
| *SNORA28* | NR_002964 | Small nucleolar RNA, H/ACA box 28, pseudouridylation |
| *STAT6* | NM_003153 | STAT6 mediates effects of interleukin 4 |
| *SUZ12* | NM_015355 | SUZ12 PRC2 subunit, histone methylation |
| *TBC1D22A* | NM_014346 | GTPase-activating, intracellular protein transport (predicted) |
| *TIMM23* | NM_006327 | Subunit of mitochondrial inner membrane translocase |
| *TMEM175* | NM_032326 | Proton channel, lysosomal pH stability, autophagy |
| *TMEM258* | NM_014206 | Oligosaccharyl transferase complex subunit |
| *ZFAND3* | NM_021943 | DNA- and zinc-binding activity (predicted) |

^1^ <https://www.ncbi.nlm.nih.gov/genbank/>

^2^ Potentially NK cell-related functions according to GeneCards ([www.genecards.org](http://www.genecards.org)) and to additional references.

^3^ Wang D et al: Silencing of lncRNA CHRM3-AS2 Expression Exerts Anti-Tumour Effects Against Glioma via Targeting microRNA-370-5p/KLF4. *Front Oncol* 2022, 12:856381.

^4^ Li S et al: Histone variant H2A.Z modulates nucleosome dynamics to promote DNA accessibility. *Nat Commun* 2023, 14(1):769. Xu Y et al: Histone H2A.Z controls a critical chromatin remodeling step required for DNA double-strand break repair. *Mol Cell* 2012, 48(5):723-733.

^5^ Hoyer MJ et al: Mechanisms Controlling Selective Elimination of Damaged Lysosomes. *Curr Opin Physiol* 2022, 29:100590.

^6^ Tannous A et al: N-linked sugar-regulated protein folding and quality control in the ER. *Semin Cell Dev Biol* 2015, 41:79-89. Takeda K, Qin SY, Matsumoto N, Yamamoto K: Association of malectin with ribophorin I is crucial for attenuation of misfolded glycoprotein secretion. *Biochem Biophys Res Commun* 2014, 454(3):436-440.

^7^ UPR: Unfolded protein response

^8^ Wang L et al: PLCbeta2 negatively regulates the inflammatory response to virus infection by inhibiting phosphoinositide-mediated activation of TAK1. *Nat Commun* 2019, 10(1):746.

^9^ Nguyen TT et al: PLEKHG3 enhances polarized cell migration by activating actin filaments at the cell front. *Proc Natl Acad Sci U S A* 2016, 113(36):10091-10096.

^10^ Sheng Y et al: RAB37 interacts directly with ATG5 and promotes autophagosome formation via regulating ATG5-12-16 complex assembly. *Cell Death Differ* 2018, 25(5):918-934.

**Supplementary Table 2** Fluorochrome-conjugated monoclonal antibodies (BD Biosciences) used
 in flow cytometry.

| **Antibody** | **Supplier** | **Catalogue number** |
| --- | --- | --- |
| BV421 Mouse Anti-Stat3 (pS727),  clone 49/p-Stat3 | BD Biosciences | 565416 |
| BV421 Mouse IgG1, k Isotype control, clone X40 | BD Biosciences | 562438 |
| AF647 Mouse Anti-Stat5 (pY694),  clone 47/Stat5(pY694) | BD Biosciences | 612599 |
| AF647 Mouse IgG1 κ Isotype control, clone MOPC-21 | BD Biosciences | 557783 |
| AF488 Mouse Anti-Human CD56 (NCAM-1), clone B159 | BD Biosciences | 561905 |
| BV510 Mouse Anti-Human CD3,  clone UCHT1 | BD Biosciences | 563109 |
| CD45 PerCP, clone 2D1 | BD Biosciences | 345809 |
| BV510 Mouse Anti-Ki-67, clone B56 | BD Biosciences | 563462 |
| BV510 Mouse IgG1, k Isotype control, clone X40 | BD Biosciences | 562946 |

**Supplementary Table 3** Demographics and clinical characteristics of the patients in the Phosflow
 and Ki-67 subcohorts.

|  | **Phosflow** | | **Ki-67** | |
| --- | --- | --- | --- | --- |
|  | Sepsis (n = 19) | SIRS (n = 11) | Sepsis (n = 33) | SIRS (n = 18) |
| **Demographics**  Age [mean ± SD (years)] | 68 ± 14 | 63 ± 12 | 66 ± 13 | 65 ± 14 |
| Male/female | 9/10 | 5/6 | 19/14 | 11/7 |
| **Septic focus [n (%)]** |  |  |  |  |
| Abdominal | 4 (21.1) |  | 9 (27.3) |  |
| Pulmonal | - |  | 3 (9.1) |  |
| Bloodstream | 6 (31.6) |  | 7 (21.2) |  |
| Urogenital | 4 (21.1) |  | 4 (12.1) |  |
| Joint | 1 (5.3) |  | 1 (3.0) |  |
| **SIRS etiology [n (%)]** |  |  |  |  |
| Abdominal surgery |  | 8 (72.7) |  | 10 (55.6) |
| Vascular surgery |  | 1 (9.1) |  | 3 (16.7) |
| Spinal surgery |  | 1 (9.1) |  | 1 (5.6) |
| Other |  | 1 (9.1)^a^ |  | 4 (22.2)^b^ |
| **SOFA score ± SD** | 7.1 ± 4.3* | 3.7 ± 1.5 | 6.4 ± 4.1 | 4.4 ± 1.8 |
| **Blood parameters [mean ± SD]** |  |  |  |  |
| WBC (10^9^/L) | 21.28 ± 15.30 | 13.97 ± 2.96 | 18.93 ±12.52 | 13.16 ± 3.55 |
| Lactate (mmol/L) (n > 2 mmol/L) | 4.12 ± 7.52* (5) | 2.03 ± 1.16 (5) | 3.65 ± 6.67 (11) | 1.73 ± 1.09 (6) |
| CRP (mg/L) | 192.05 ± 87.16** | 103.08 ± 51.72 | 204.9 ± 108.1** | 87.06 ± 54.76 |

^a^ Bleeding leg ulcer.

^b^ Other SIRS eitiologies were once each gastrointestinal bleeding, craniocerebral trauma, bleeding leg
 ulcer, and split-sking graft.

* P < 0.05 for sepsis versus SIRS after the t-test.

** P < 0.005 for sepsis versus SIRS after the t-test.

Abbreviations: CRP: C-reactive protein; SD: Standard deviation; SIRS: Systemic inflammatory response syndrome; SOFA: Sequential organ failure assessment; WBC: white blood cell

**Supplementary Table 4** KEGG pathway enrichment analysis in NK cells from patients with sepsis, SIRS, and presurgical controls (GSE123730).

| **Normalized enrichment score^a^** | | |  |
| --- | --- | --- | --- |
| **Sepsis vs. Presurgical** | **SIRS vs. Presurgical** | **Sepsis vs. SIRS** | **KEGG pathway^b^** |
|  |  |  | **Metabolism** |
| **3.11** | **1.69** | **2.83** | oxidative phosphorylation |
| **2.23** | **1.67** | **1.72** | biosynthesis of cofactors |
| **2.28** | **1.89** | 1.48 | fatty acid metabolism |
| **2.14** | **1.95** | 1.03 | fatty acid elongation |
| **1.94** | **1.89** | 1.09 | fatty acid degradation |
| **2.10** | **1.75** | 1.23 | valine, leucine and isoleucine degradation |
|  |  |  | N-glycan biosynthesis (1): |
| **1.86** | **1.77** | 1.22 | N-glycan biosynthesis |
| **1.76** | **1.94** | 0.99 | various types of N-glycan biosynthesis |
| **2.26** | 1.65 | **1.87** | cysteine and methionine metabolism |
| **2.10** | 1.04 | **2.15** | nucleotide metabolism |
| **2.04** | -0.85 | **2.32** | pyrimidine metabolism |
| **1.94** | **1.32** | **1.74** | purine metabolism |
| **2.08** | 1.20 | **1.94** | pentose phosphate pathway |
| **2.05** | 1.24 | **1.73** | glutathione metabolism |
| **2.04** | 1.59 | **1.71** | pyruvate metabolism |
| **2.00** | 1.05 | **1.93** | sulfur metabolism |
| **1.99** | 1.44 | **1.52** | biosynthesis of amino acids |
| **1.83** | 1.17 | **1.62** | drug metabolism - other enzymes |
| **1.75** | 1.21 | **1.68** | amino sugar and nucleotide sugar metabolism |
| **1.75** | 1.27 | **1.59** | glycolysis / gluconeogenesis |
| **1.99** | 1.33 | 1.63 | citrate cycle |
| **1.93** | 1.22 | 1.59 | one carbon pool by folate |
| **1.70** | 1.35 | 1.51 | fructose and mannose metabolism |
|  |  |  | glycosaminoglycan biosynthesis (2): |
| 1.33 | **1.98** | -1.09 | glycosaminoglycan biosynthesis - chondroitin sulfate / dermatan sulfate |
| 1.57 | **1.89** | 0.68 | glycosaminoglycan biosynthesis - heparan sulfate / heparin |
| 1.27 | **1.58** | -1.01 | glycerophospholipid metabolism |
| 1.44 | -1.11 | **1.80** | terpenoid backbone biosynthesis |
| 1.61 | 1.10 | **1.72** | biosynthesis of nucleotide sugars |
|  |  |  | **Genetic information processing** |
|  |  |  | protein production (3): |
| **3.24** | **2.21** | **2.47** | ribosome |
| **2.59** | **1.83** | **1.88** | protein processing in endoplasmic reticulum |
| **2.49** | **1.73** | **2.09** | aminoacyl-tRNA biosynthesis |
| **2.43** | **1.70** | **2.65** | protein export |
| **2.43** | **1.62** | **2.02** | ribosome biogenesis in eukaryotes |
| **1.76** | **1.91** | 0.88 | RNA polymerase |
| **2.84** | 0.98 | **2.75** | proteasome |

**Supplementary Table 4 continued**

| **Normalized enrichment score^a^** | | |  |
| --- | --- | --- | --- |
| **Sepsis vs. Presurgical** | **SIRS vs. Presurgical** | **Sepsis vs. SIRS** | **KEGG pathway^b^** |
|  |  |  | **Genetic information processing** |
| **1.82** | -0.49 | **2.12** | DNA replication |
|  |  |  | DNA repair (4): |
| **1.92** | -1.30 | **2.25** | mismatch repair |
| **1.90** | 0.79 | **1.87** | base excision repair |
| **1.80** | -1.42 | **2.26** | homologous recombination |
| **1.77** | 0.81 | **2.08** | nucleotide excision repair |
| **1.70** | -1.34 | **2.14** | Fanconi anemia pathway |
| **1.73** | -1.20 | **1.73** | nucleocytoplasmic transport |
| **1.52** | -0.94 | 1.44 | spliceosome |
|  |  |  | **Environmental information processing, Cellular processes, Organismal systems** |
| **2.07** | **1.93** | 1.22 | antigen processing and presentation |
| **1.70** | **1.81** | 0.92 | hematopoietic cell lineage |
|  |  |  | phagosome / lysosome (5): |
| **1.75** | **1.66** | 1.23 | phagosome |
| **1.48** | 1.40 | 1.30 | lysosome |
| **2.48** | -1.29 | **2.83** | cell cycle |
| **1.49** | 1.20 | **1.48** | cellular senescence |
| **-1.93** | -1.16 | **-1.85** | neuroactive ligand-receptor interaction |
| **1.73** | 1.15 | 1.24 | peroxisome |
| **1.50** | 1.38 | 0.99 | HIF-1 signaling pathway |
|  |  |  | cell adhesion (6): |
| 1.04 | **1.87** | -1.35 | adherens junction |
| -0.90 | **1.51** | -1.27 | cell adhesion molecules |
| 2.28 | **1.65** | 1.55 | ferroptosis |
| 1.10 | **1.63** | -1.31 | phospholipase D signaling pathway |
|  |  |  | inflammatory signaling pathways (7): |
| -0.77 | **-1.57** | 1.22 | TNF signaling pathway |
| -0.93 | **-1.61** | 0.84 | NF-kappa B signaling pathway |
| -1.36 | **-1.79** | 0.91 | C-type lectin receptor signaling pathway |
|  |  |  | microbial pattern recognition receptors (8): |
| 0.95 | **-1.57** | **1.54** | RIG-I-like receptor signaling pathway |
| -0.93 | **-1.99** | **1.79** | NOD-like receptor signaling pathway |
| 1.18 | -1.32 | 1.50 | Necroptosis |
|  |  |  | cyclic nucleotide signaling (9): |
| -1.31 | -0.97 | **-1.45** | cAMP signaling pathway |
| -1.35 | 1.27 | **-1.58** | cGMP-PKG signaling pathway |

^a^ Bold print indicates a false discovery rate-q value < 0.05 (statistically significant enrichment). The complete KEGG pathway enrichment analysis results are available from heiDATA (https://heidata.uni-heidelberg.de/previewurl.xhtml?token=f99c5b81-347a-4a6b-9fff-7a96108aabe9).

^b^ Postposed numbers in parentheses indicate functional groups of pathways and correspond to the
 numbers used in Figure 1 of the main manuscript.

**Supplementary Table 5** Enrichment analysis of immune signaling-related Gene Ontology (GO) biological processes in NK cells from patients with sepsis, SIRS, and presurgical controls (GSE123730).

| **Normalized enrichment score^a^** | | |  |
| --- | --- | --- | --- |
| **Sepsis vs. Presurgical** | **SIRS vs. Presurgical** | **Sepsis vs. SIRS** | **GO biological process** |
|  |  |  | immune response general |
| **1.39** | 1.21 | **1.32** | regulation of immune response |
| **1.41** | 0.00 | **1.33** | positive regulation of immune response |
| **1.35** | 0.00 | 1.35 | immune response-activating signaling pathway |
| 0.00 | 0.00 | **1.46** | positive regulation of inflammatory response |
|  |  |  | leukocyte |
| **1.54** | 0.00 | **1.44** | leukocyte mediated immunity |
| **1.29** | **1.52** | 0.00 | leukocyte activation |
| **1.24** | **1.42** | 0.00 | regulation of leukocyte activation |
| 1.24 | **1.44** | 0.00 | positive regulation of leukocyte activation |
|  |  |  | lymphocyte |
| **1.32** | **1.60** | 0.00 | lymphocyte activation |
| **1.60** | 0.00 | **1.61** | lymphocyte mediated immunity |
| 0.00 | **1.53** | 0.00 | regulation of lymphocyte activation |
| 0.00 | **1.56** | 0.00 | positive regulation of lymphocyte activation |
|  |  |  | innate immune response |
| 0.00 | 0.00 | **1.28** | innate immune response |
| 0.00 | 0.00 | **1.35** | activation of innate immune response |
| 0.00 | 0.00 | **1.33** | positive regulation of innate immune response |
| 0.00 | 0.00 | **1.31** | regulation of innate immune response |
|  |  |  | immune effector process general |
| **1.49** | 1.28 | **1.28** | immune effector process |
| **1.59** | 0.00 | **1.59** | production of molecular mediator of immune response |
| **1.80** | 0.00 | **1.94** | regulation of T-helper 1 type immune response |
|  |  |  | cellular cytotoxicity |
| **1.43** | 0.00 | **1.47** | cell killing |
| 1.44 | 0.00 | **1.76** | killing of cells of another organism |
| **1.23** | 0.00 | **1.42** | cellular response to cytokine stimulus |
|  |  |  | cytokine production |
| 0.00 | **-1.34** | **1.33** | cytokine production |
| 0.00 | **-1.35** | **1.37** | regulation of cytokine production |
| **1.28** | 0.00 | **1.35** | positive regulation of cytokine production |
| **1.44** | 0.00 | 0.00 | regulation of cytokine production involved in immune response |
| 0.00 | **-1.64** | **1.36** | negative regulation of cytokine production |
| 0.00 | **-1.80** | **1.68** | regulation of type I interferon production |
| 0.00 | **-1.93** | **1.64** | positive regulation of type I interferon production |
| 0.00 | **-1.88** | 0.00 | positive regulation of interferon-alpha production |
| 0.00 | **-1.87** | 1.56 | interferon-beta production |
|  |  |  | response to cytokine |
| 0.00 | **-1.44** | 0.00 | response to tumor necrosis factor |
| 0.00 | 0.00 | **1.82** | response to interferon-beta |
| 0.00 | **-1.56** | 0.00 | response to type II interferon |
| 0.00 | **-1.62** | 0.00 | cellular response to type II interferon |
|  |  |  | cytokine mediated signaling pathway |
| 0.00 | **-1.60** | **1.37** | cytokine-mediated signaling pathway |
| 0.00 | **-1.60** | 1.49 | negative regulation of cytokine-mediated signaling pathway |
| 0.00 | **-1.66** | **1.63** | tumor necrosis factor-mediated signaling pathway |
| 0.00 | 0.00 | **-1.70** | positive regulation of transforming growth factor beta receptor signaling pathway |
| 0.00 | **-1.70** | 1.53 | interferon-mediated signaling pathway |
| 0.00 | **-2.20** | 0.00 | type II interferon-mediated signaling pathway |
|  |  |  | NF-kappaB |
| 0.00 | 0.00 | **1.43** | canonical NF-kappaB signal transduction |
| 0.00 | 0.00 | **1.50** | positive regulation of NF-kappaB transcription factor activity |
| 0.00 | 0.00 | **1.44** | positive regulation of canonical NF-kappaB signal transduction |
|  |  |  | pattern recognition receptor |
| 0.00 | 0.00 | **1.40** | pattern recognition receptor signaling pathway |
| 0.00 | 0.00 | **1.80** | positive regulation of pattern recognition receptor signaling pathway |
| 0.00 | 0.00 | **1.45** | cytoplasmic pattern recognition receptor signaling pathway |
| 0.00 | 0.00 | **1.50** | regulation of cytoplasmic pattern recognition receptor signaling pathway |
| **-1.50** | 0.00 | **-1.77** | adenylate cyclase-modulating G protein-coupled receptor signaling pathway |
|  |  |  |  |
| ^a^ Bold print indicates a false discovery rate-q value < 0.05 (statistically significant enrichment). The complete GO term enrichment analysis results are available from heiDATA (https://heidata.uni-heidelberg.de/previewurl.xhtml?token=f99c5b81-347a-4a6b-9fff-7a96108aabe9). | | | |

**Supplementary Table 6** Enrichment analysis of additional Gene Ontology (GO) terms in NK cells from patients with sepsis, SIRS, and presurgical controls (GSE123730).

| **Normalized enrichment score^a^** | | |  |
| --- | --- | --- | --- |
| **Sepsis vs. Presurgical** | **SIRS vs. Presurgical** | **Sepsis vs. SIRS** | **GO term^b^** |
|  |  |  | **GO biological process** |
| **2.16** | **1.68** | **1.49** | response to unfolded protein |
| 0.00 | 0.00 | **1.80** | pyroptosis |
|  |  |  | ncRNA processing (1): |
| **2.36** | **1.95** | **1.64** | ncRNA processing |
| **2.15** | **1.89** | **1.48** | ncRNA metabolic process |
| 1.58 | 0.00 | **1.69** | snRNA metabolic process |
|  |  |  | gene silencing (2): |
| 0.00 | **-2.10** | 0.00 | miRNA-mediated gene silencing by mRNA destabilization |
| 0.00 | **-2.02** | **1.47** | post-transcriptional gene silencing |
| 0.00 | **-2.01** | **1.45** | miRNA-mediated post-transcriptional gene silencing |
| 0.00 | **-1.96** | **1.46** | regulatory ncRNA-mediated gene silencing |
| 0.00 | **-1.60** | **1.46** | regulatory ncRNA-mediated post-transcriptional gene silencing |
|  |  |  | small GTPase signaling (3) |
| 0.00 | 0.00 | **-1.40** | regulation of small GTPase mediated signal transduction |
| 0.00 | **1.33** | **-1.33** | small GTPase-mediated signal transduction |
|  |  |  | microtubule cytoskeleton (4): |
| **2.17** | 0.00 | **1.93** | microtubule cytoskeleton organization |
| **1.35** | 0.00 | **1.80** | microtubule-based process |
|  |  |  | membrane ruffling (5): |
| **2.09** | 0.00 | **1.78** | regulation of ruffle assembly |
| **1.99** | 0.00 | **1.67** | ruffle assembly |
| **1.72** | 0.00 | 0.00 | ruffle organization |
|  |  |  | actin nucleation (6): |
| **1.91** | 0.00 | 0.00 | Arp2/3 complex-mediated actin nucleation |
| **1.65** | 0.00 | 0.00 | actin nucleation |
| **1.57** | 0.00 | 0.00 | membrane invagination |
| **1.35** | 0.00 | 1.22 | regulation of cytoskeleton organization |
|  |  |  | actin cytoskeleton (7): |
| 0.00 | 0.00 | **-1.36** | actin filament-based process |
| 0.00 | 0.00 | **-1.35** | actin cytoskeleton organization |
| 0.00 | 0.00 | **-1.39** | cell junction organization |
|  |  |  | cell morphogenesis (8): |
| **-1.30** | 0.00 | **-1.39** | cell projection morphogenesis |
| 0.00 | 0.00 | **-1.33** | cell morphogenesis |
| 0.00 | 0.00 | **-1.45** | regulation of cell morphogenesis |
|  |  |  |  |
| **Supplementary Table 6 continued** | | | |
| **Normalized enrichment score^a^** | | |  |
| **Sepsis vs. Presurgical** | **SIRS vs. Presurgical** | **Sepsis vs. SIRS** | **GO term^b^** |
|  |  |  | **GO biological process** |
|  |  |  | vesicle biogenesis, coating, budding (9): |
| **2.17** | **1.81** | **1.72** | COPII-coated vesicle budding |
| **2.04** | **1.82** | 1.66 | COPII vesicle coating |
| **1.92** | 1.70 | 1.55 | vesicle coating |
| **1.92** | **1.65** | 1.57 | vesicle budding from membrane |
|  |  |  | Golgi vesicle transport (10): |
| **2.13** | **2.15** | 0.00 | retrograde vesicle-mediated transport, Golgi to endoplasmic reticulum |
| **2.04** | **1.82** | 1.66 | vesicle targeting, rough ER to cis-Golgi |
| **1.81** | **1.63** | 0.00 | Golgi vesicle transport |
|  |  |  | ADP-ribosylation factor (ARF) protein (11): |
| **-1.75** | 0.00 | **-1.92** | ARF protein signal transduction |
| **-1.75** | 0.00 | **-1.92** | regulation of ARF protein signal transduction |
|  |  |  | **GO cellular compartment** |
|  |  |  | Ribonucleoprotein (12): |
| **2.11** | **1.79** | 1.65 | sno(s)RNA-containing ribonucleoprotein complex |
| **1.45** | 1.31 | 0.00 | ribonucleoprotein granule |
| **1.22** | 1.19 | **1.23** | nuclear body |
| 0.00 | **-2.17** | **1.56** | RISC complex |
|  |  |  | Golgi vesicle transport (13): |
| **2.04** | **1.86** | 0.00 | endoplasmic reticulum-Golgi intermediate compartment membrane |
| **2.00** | **1.83** | 0.00 | COPII-coated ER to Golgi transport vesicle |
| **1.82** | **1.81** | 0.00 | ER to Golgi transport vesicle membrane |
| **1.42** | **1.62** | 0.00 | Golgi membrane |
|  |  |  | coated vesicle (14): |
| **2.15** | **1.95** | 0.00 | COPI-coated vesicle |
| **1.62** | **1.80** | 0.00 | coated vesicle membrane |
| **1.36** | **1.60** | 0.00 | coated vesicle |
|  |  |  | clathrin-mediated endocytosis (15): |
| 0.00 | **2.09** | -1.41 | clathrin-coated endocytic vesicle membrane |
| 0.00 | **1.68** | 0.00 | clathrin-coated vesicle membrane |
| 0.00 | **1.64** | 0.00 | clathrin-coated endocytic vesicle |
| 0.00 | **1.48** | 0.00 | endocytic vesicle membrane |
| 0.00 | **1.45** | 0.00 | transport vesicle membrane |
| 1.23 | **1.37** | 0.00 | transport vesicle |
| 0.00 | **1.36** | 0.00 | clathrin-coated vesicle |
| ^a^ Bold print indicates a false discovery rate-q value < 0.05 (statistically significant enrichment). The complete GO term enrichment analysis results are available from heiDATA (https://heidata.uni-heidelberg.de/previewurl.xhtml?token=f99c5b81-347a-4a6b-9fff-7a96108aabe9).  ^b^ Postposed numbers in parentheses indicate functional groups and correspond to the   numbers used in Figure 2 of the main manuscript and in Suppl. Figures 4 and 5. | | | |


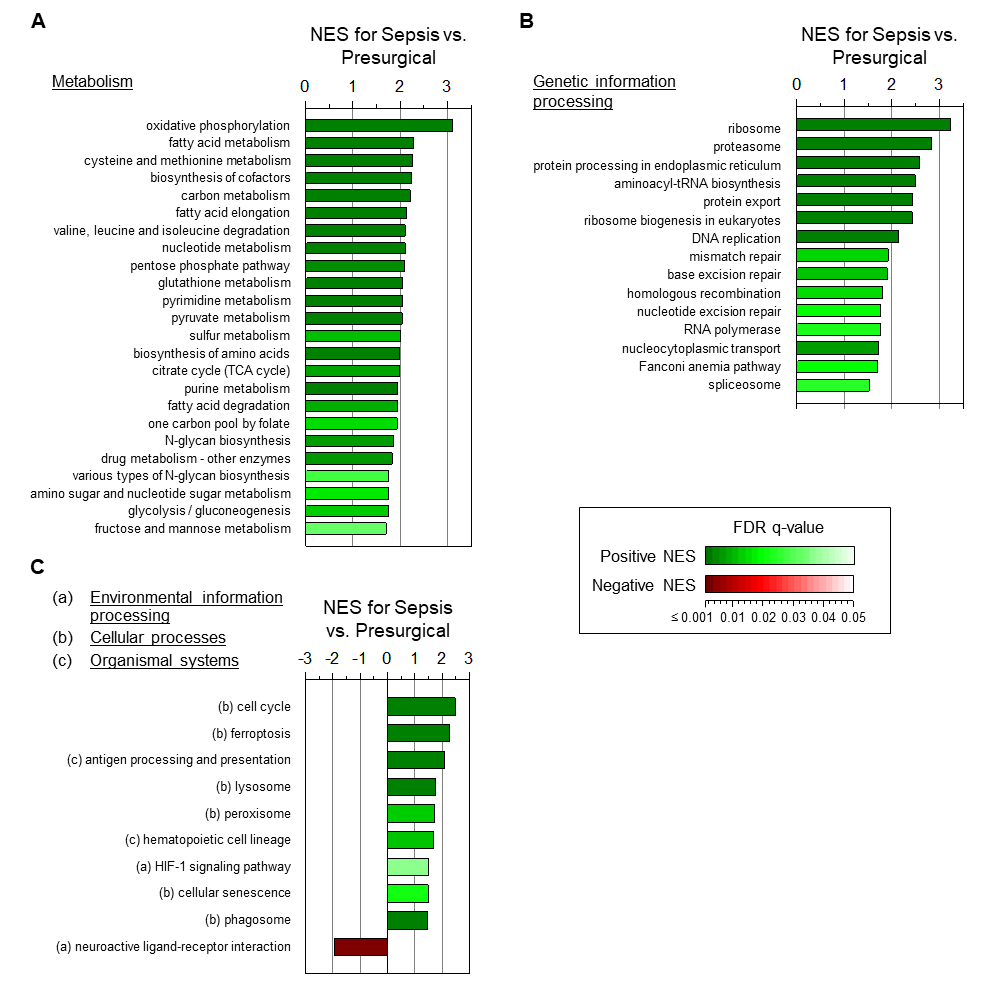


**Supplementary Figure 1. KEGG pathway enrichment analysis in NK cells from patients with sepsis and presurgical controls (GSE123730).** NK cells were from patients with sepsis on ICU admission (n = 10) and presurgical controls (n = 19). Panels **A**–**C** display the normalized enrichment score (NES) values for NK cell-attributable KEGG pathways with a false discovery rate (FDR)-q value < 0.05 grouped by KEGG pathway categories.


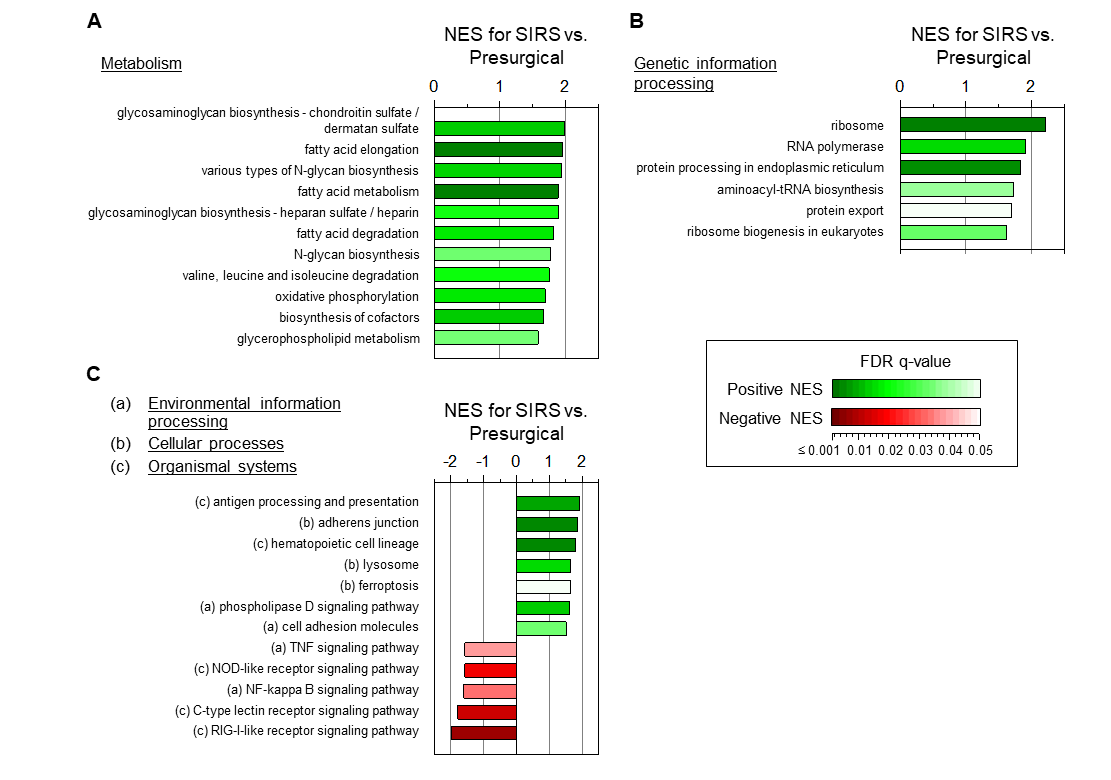


**Supplementary Figure 2. KEGG pathway enrichment analysis in NK cells from patients with SIRS compared to presurgical controls (GSE123730).** NK cells were from patients with SIRS on ICU admission (n = 16) and presurgical controls (n = 19). Panels **A**–**C** display the normalized enrichment score (NES) values for NK cell-attributable KEGG pathways with a false discovery rate (FDR)-q value < 0.05 grouped by KEGG pathway category.


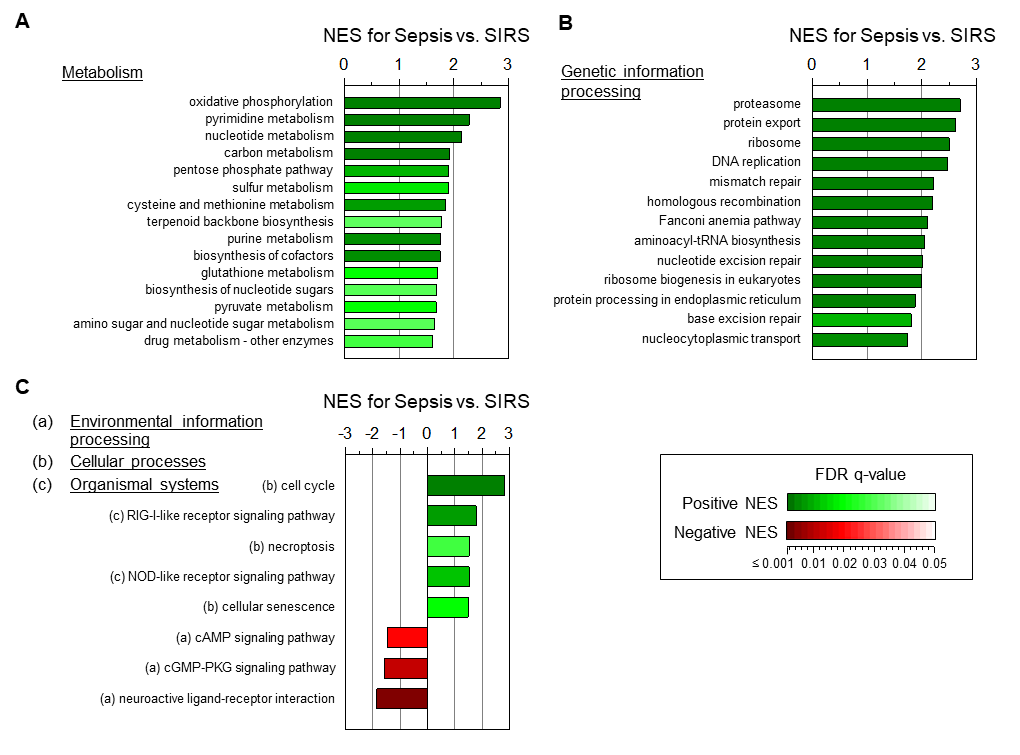


**Supplementary Figure 3. KEGG pathway enrichment analysis in NK cells from patients with sepsis compared to SIRS (GSE123730).** NK cells were from patients with sepsis (n = 10) and SIRS (n = 16) on ICU admission. Panels **A**–**C** display the normalized enrichment score (NES) values for NK cell-attributable KEGG pathways with a false discovery rate (FDR)-q value < 0.05 grouped by KEGG pathway category.


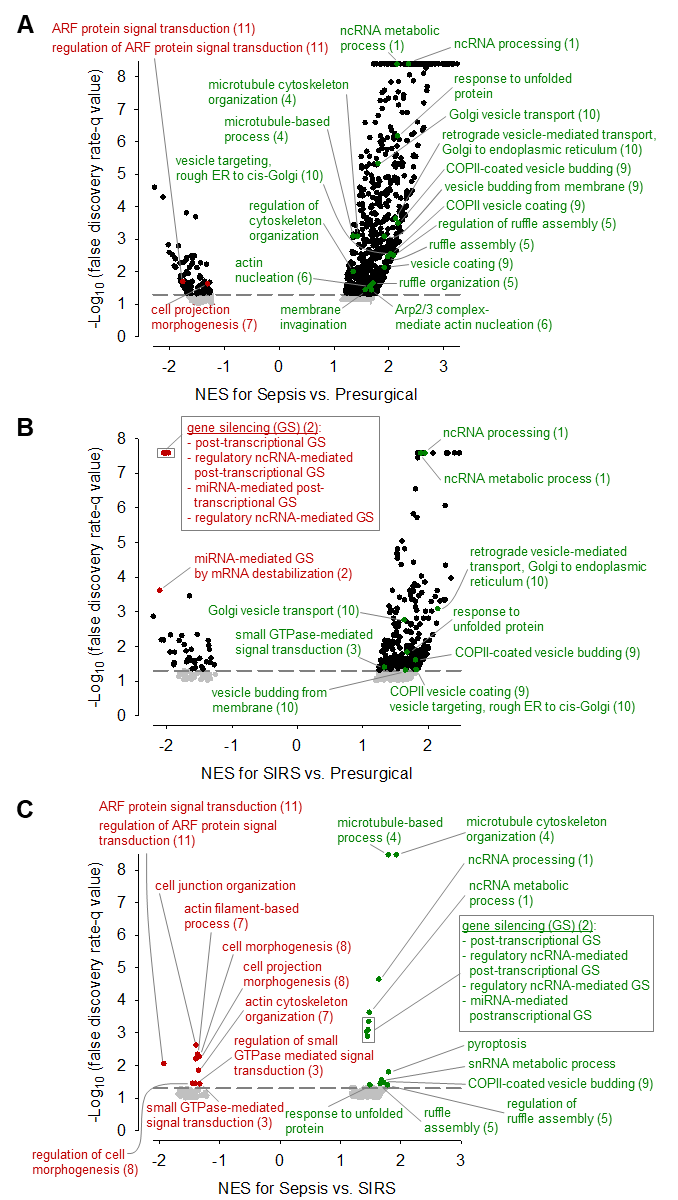


**Supplementary Figure 4. Enrichment analysis of Gene Ontology (GO) biological processes in NK cells from patients with sepsis, SIRS, and presurgical controls (GSE123730).** Volcano plots highlighting the process associations summarized in Figure 2 of the main manuscript. **A** (sepsis vs. presurgical), **B** (SIRS vs. presurgical), **C** (sepsis vs. SIRS), dashed lines (statistical significance threshold (FRD-q = 0.05)), numbers in parentheses (functional group memberships in accordance with Figure 2 and Suppl. Table 6). Processes in surrounding boxes are listed by ascending FDR values.


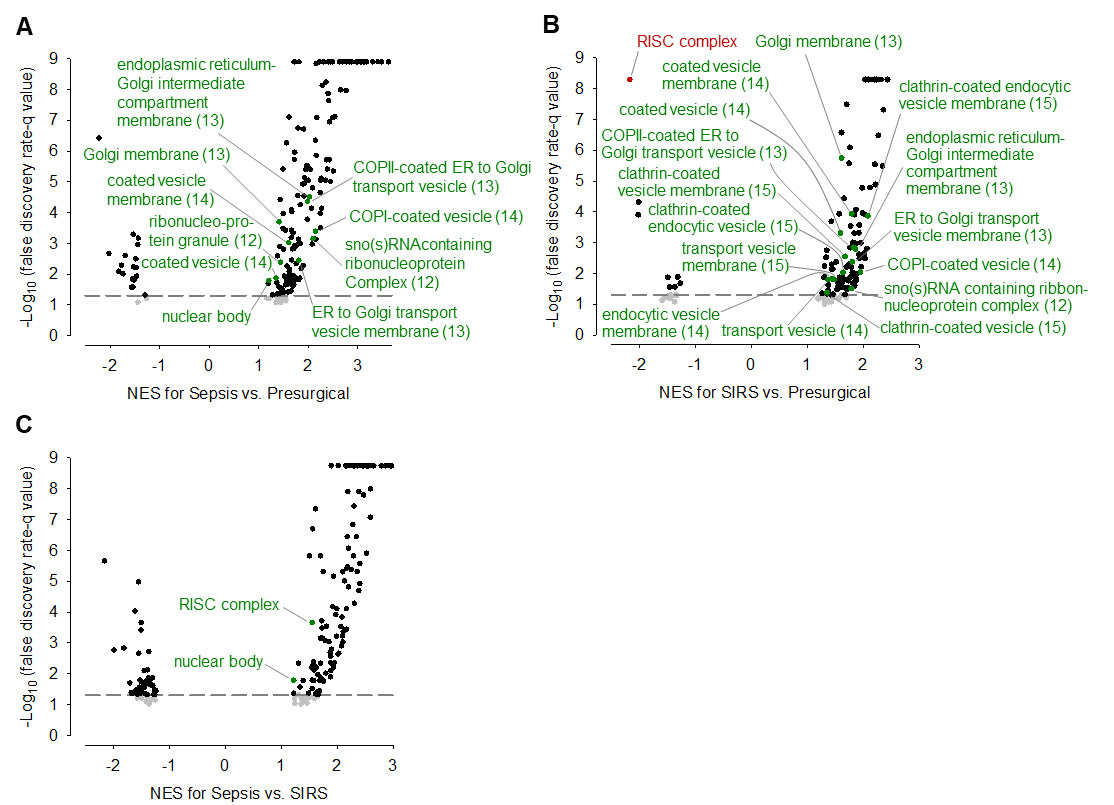


**Supplementary Figure 5. Enrichment analysis of Gene Ontology (GO) cellular compartments in NK cells from patients with sepsis, SIRS, and presurgical controls (GSE123730).** Volcano plots highlighting the compartment associations summarized in Figure 2 of the main manuscript. **A** (sepsis vs. presurgical), **B** (SIRS vs. presurgical), **C** (sepsis vs. SIRS), dashed lines (statistical significance threshold (FRD-q = 0.05)), numbers in parentheses (functional group memberships in accordance with Figure 2 and Suppl. Table 6).

**Log_2_ mean difference**

**FDR**

**Gene**

**FDR**

**Log_2_ mean difference**

**Gene**

**Log_2_ mean difference**

**FDR**

**Supplementary Figure 6.** Differentially expressed genes in NK cells from patients with sepsis and presurgical controls with a mean difference ≤ twofold and a false discovery rate (FDR) between 0.01 and 0.5 (GSE123730).

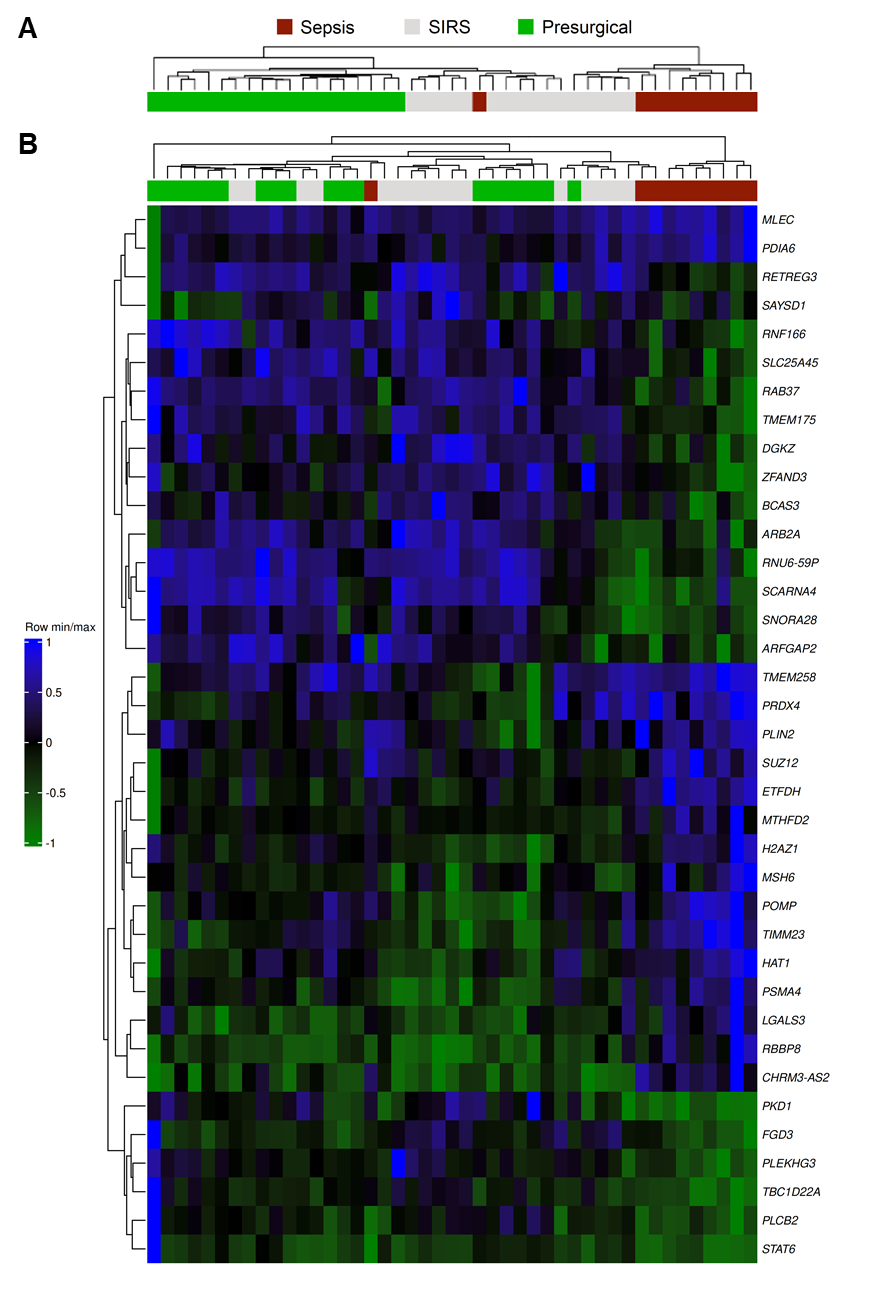


**Supplementary Figure 7. Heatmap of microarray results (GSE123730). A** Clustering result for all 208 differentially expressed genes (DEGs). **B** Clustered heatmap based on 37 DEGs selected for the determination of sepsis-SIRS differences by QuantiGene Plex. Sepsis (n = 10), SIRS (n = 16), presurgical (n = 19). The map was prepared with the ComplexHeatmap package (Gu Z. Imeta 2022, 1(3):e43.).


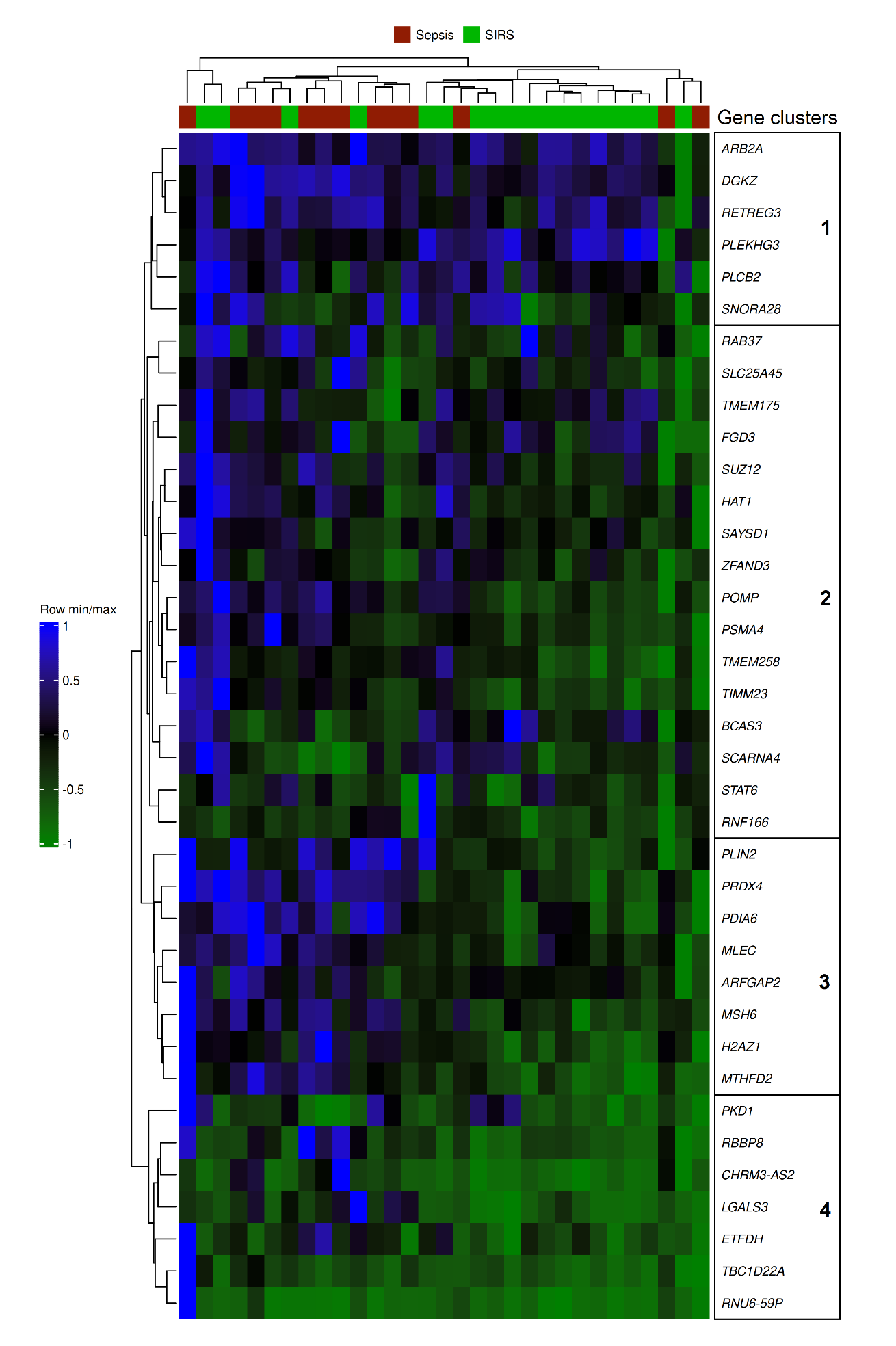


**Supplementary Figure 8. Heatmap of QuantiGene Plex results.** Sepsis (n = 15), SIRS (n = 18). The map was prepared with the ComplexHeatmap package (Gu Z. Imeta 2022, 1(3):e43.).


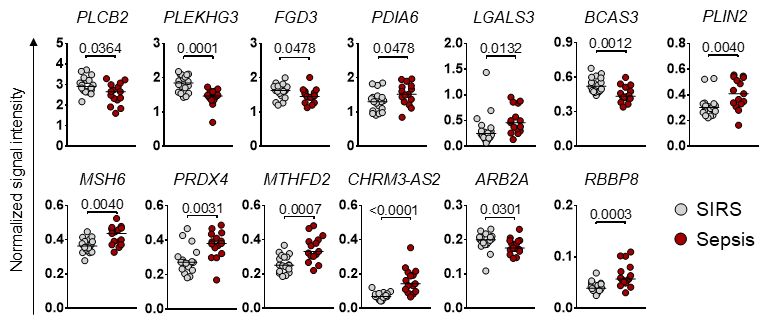


**Supplementary Figure 9. NK cell sepsis signature genes with significant sepsis-SIRS differences in the validation cohort.** QuantiGene Plex results are shown as dot plots with medians and unadjusted p-values from the Mann-Whitney U test. Sepsis (n = 15), SIRS (n = 18).


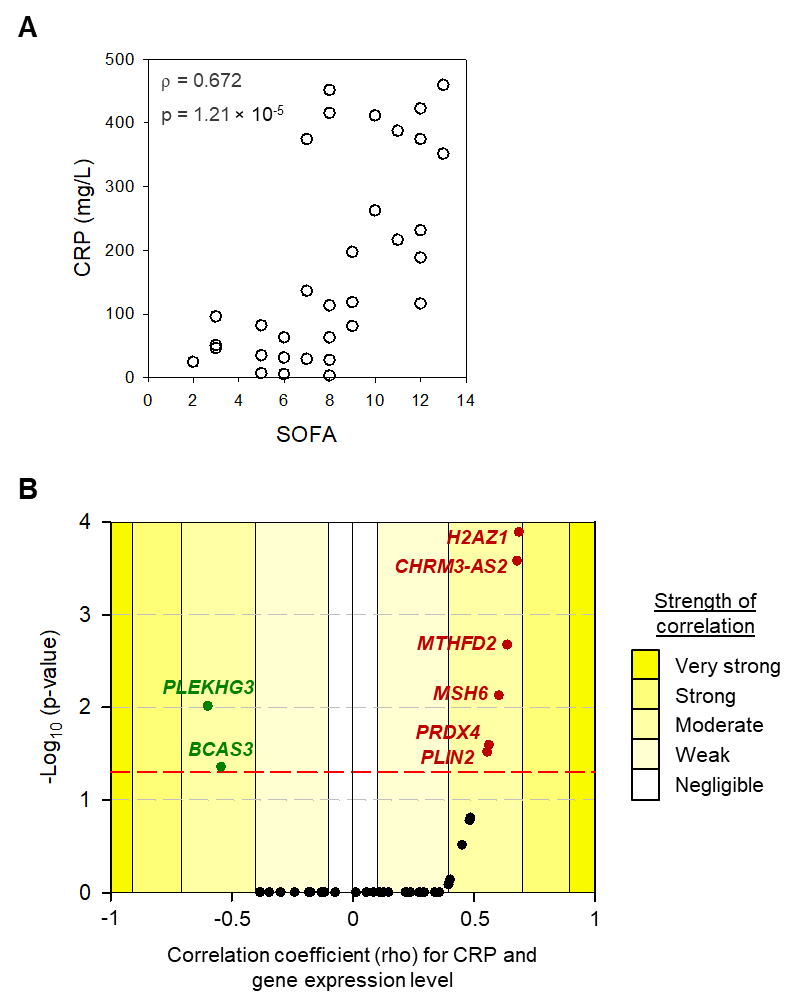


**Supplementary Figure 10. Correlations for blood C-reactive protein (CRP) in the validation cohort. A** Dot plot with Spearman's correlation coefficient (ρ) and p-value for the correlation of CRP with SOFA. **B** Correlations of CRP with the expression levels of NK cell sepsis signature genes based on QuantiGene Plex. The Bonferroni-adjusted p-value is plotted against the Spearman’s correlation coefficient with the interpretation of the correlation strength accoding to Schober et al. (2018, Anesth Analg 126, 1763-1768) indicated by yellow background shading. The dashed red line marks the threshold for statistical significance (p = 0.05). Genes with negative and positive correlations are printed in green and red, respectively. Validation cohort (n = 33).


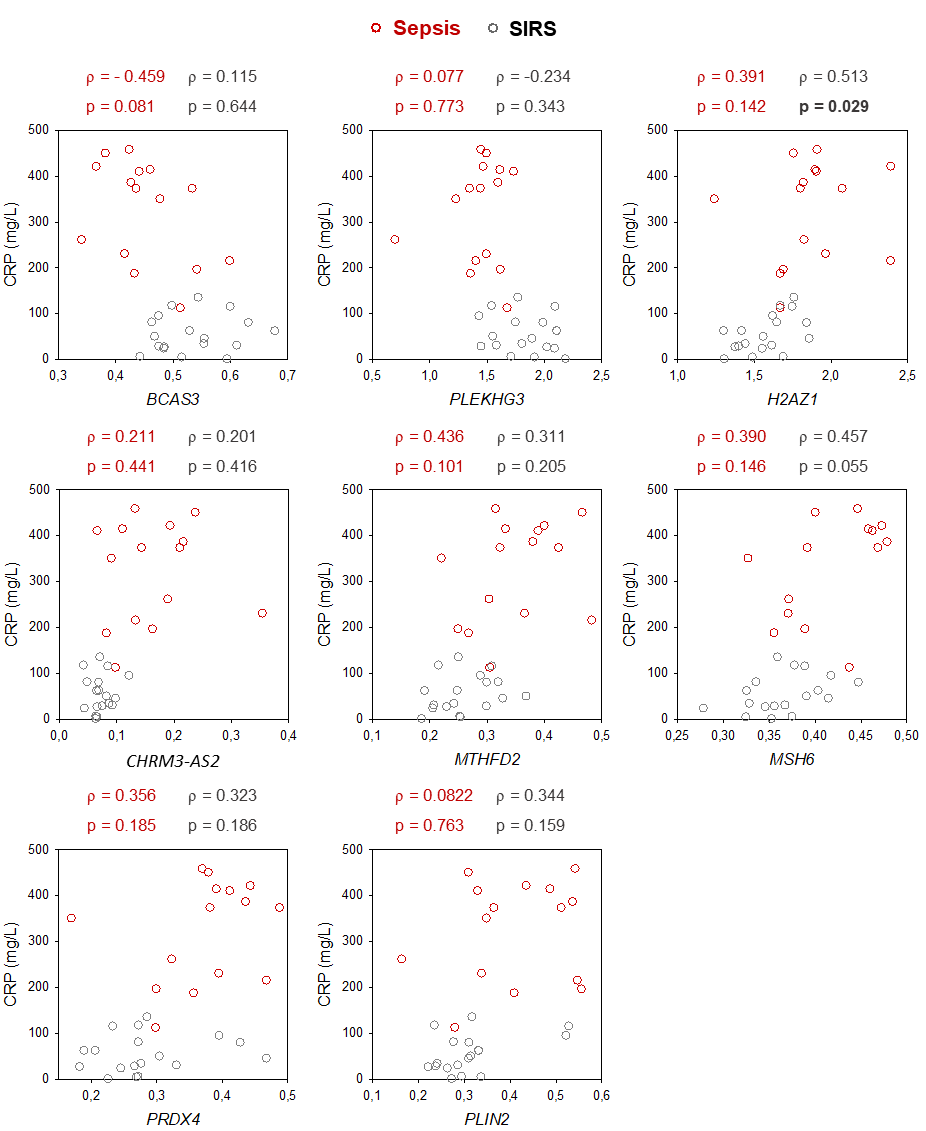


**Supplementary Figure 11. Correlations between blood C-reactive protein (CRP) and NK cell sepsis signature genes in the validation cohort.** Dot plots and Spearman's correlation coefficients (ρ) and unadjusted p-values are shown for all QuantiGene Plex results that showed significant correlations with CRP in the entire validation cohort (Fig. 4C of the main manuscript) separately for sepsis (n = 15) and SIRS (n = 18).

**
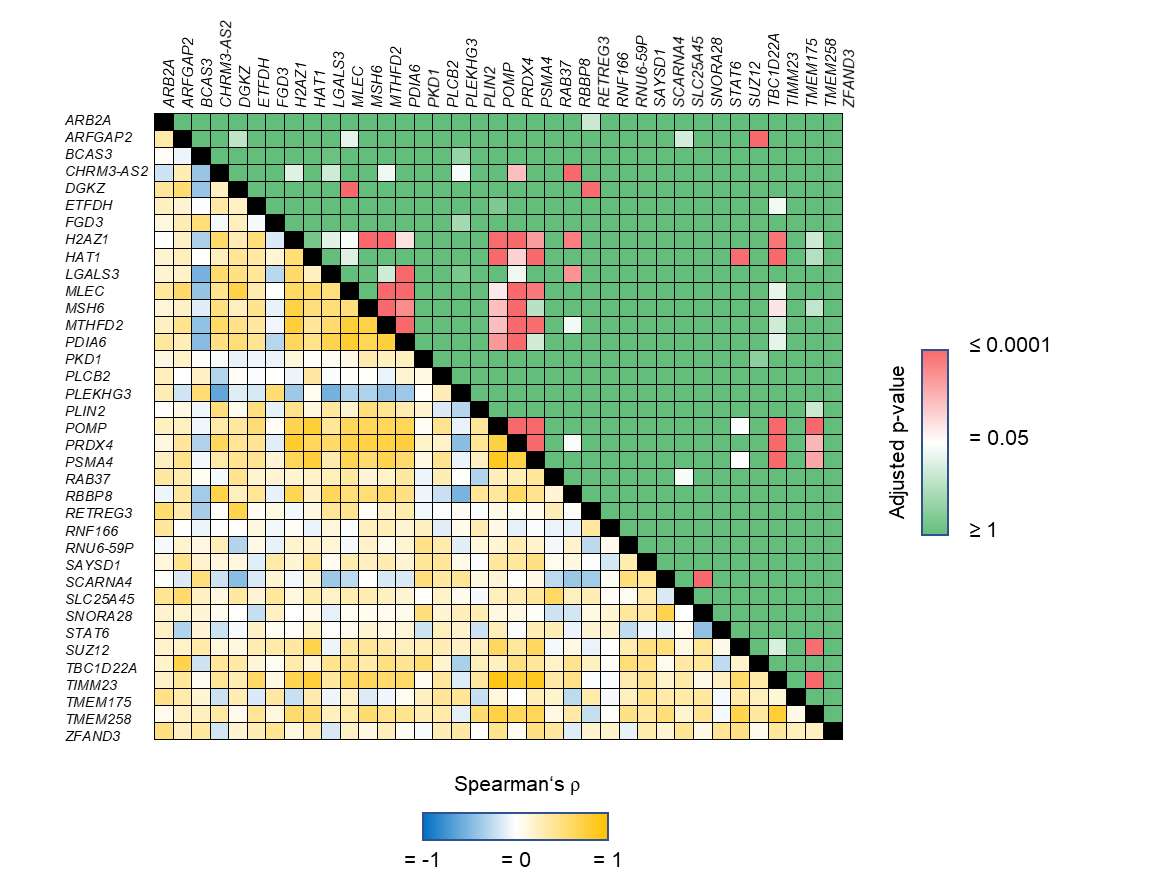
**

**Supplementary Figure 12. Intergene correlations for the NK cell sepsis gene signature in the validation cohort.** Spearman's ρ and Bonferroni-adjusted p-values for the QuantiGene Plex results (cf. Suppl. Fig. 7). Genes are ordered alphabetically by gene name. Validation cohort (n = 33).


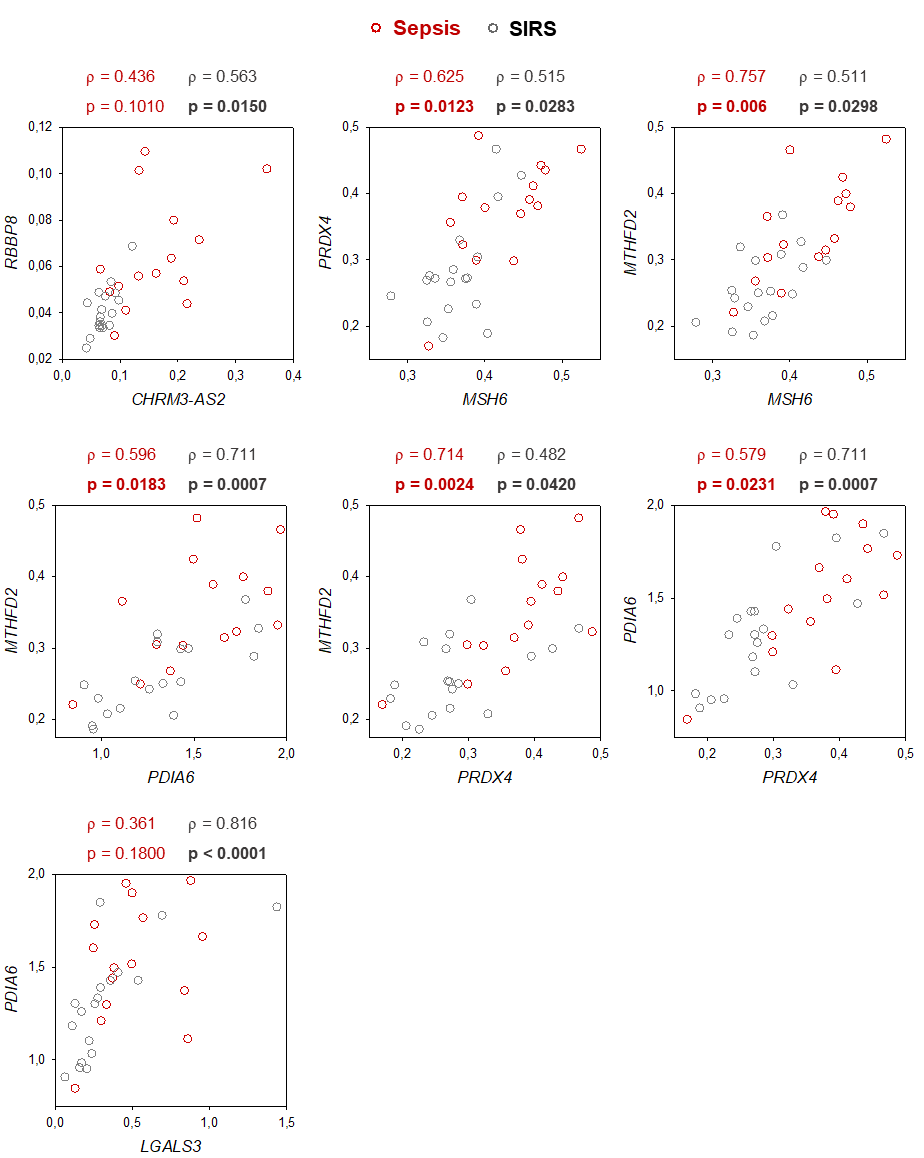


**Supplementary Figure 13. Strong intergene correlations for genes with confirmed expression level differences in sepsis and SIRS NK cells in the validation cohort.** Dot plots and the values for Spearman's ρ and unadjusted p-values. Sepsis (n = 15), SIRS (n = 18).


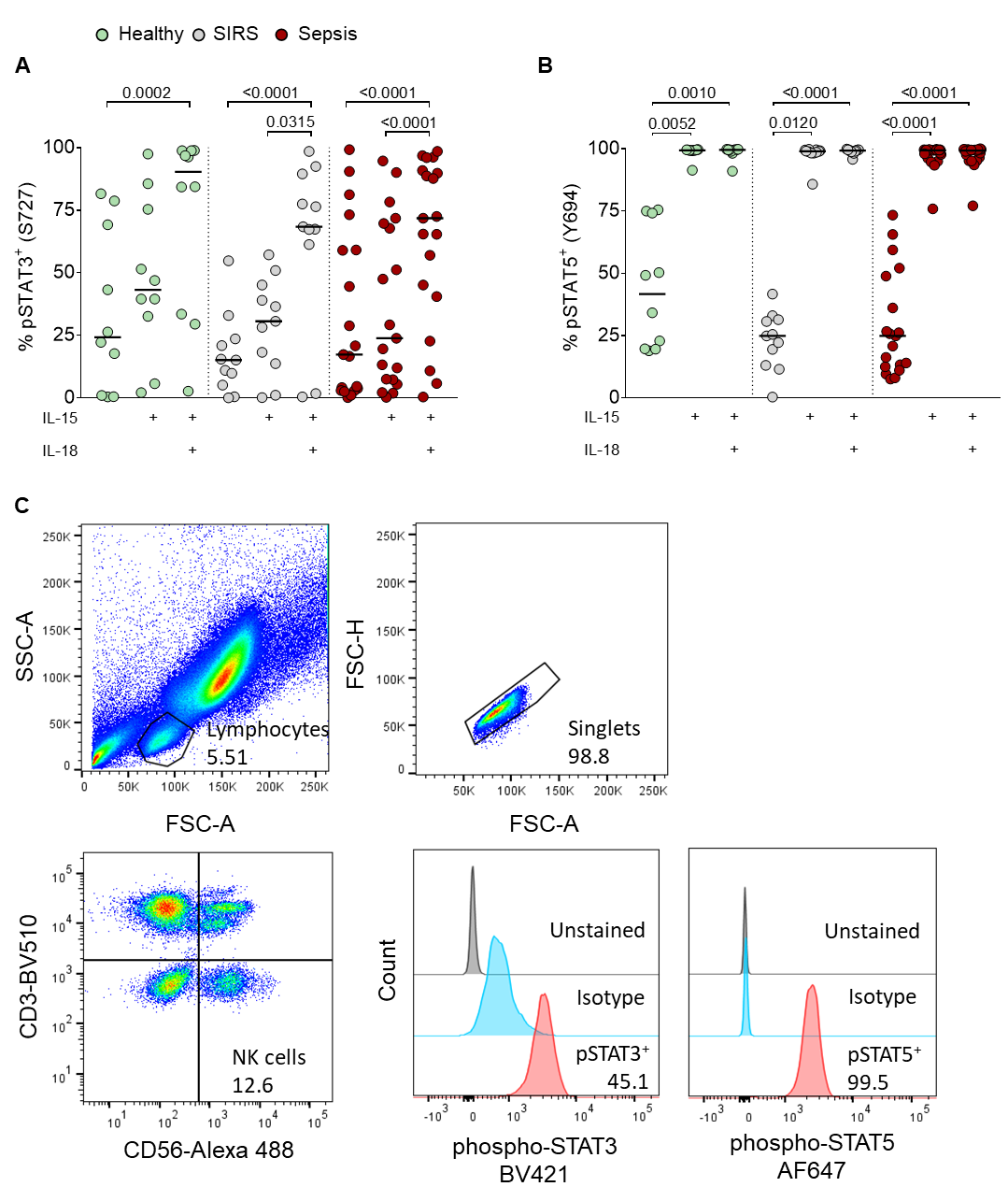


**Supplementary Figure 14.** **Quantification and representative flow cytometry analysis of STAT3 and STAT5 phosphorylation in healthy donors and patients with sepsis or SIRS.** The proportions of CD3^−^CD56^+^ NK cells positive for pSTAT3 (**A**) and pSTAT5 (**B**) were determined by Phosflow in whole blood from healthy donors (n = 10) and patients with sepsis (n = 19) or SIRS (n = 11). The anticoagulated blood was treated for 15 minutes with cytokines as indicated. P-values for the treatment effects are from the Wilcoxon signed-rank test. Patient subgroup differences for each of the given treatment conditions did not reach statistical significance according to the Mann-Whitney U test. (**C**) Representative flow cytometry plots illustrating the gating strategy (lymphocytes → singlets → CD3^-^CD56^+^ NK cells) and histograms showing unstained, isotype control, and pSTAT3 or pSTAT5 staining in the NK cell gate. Numbers indicate percentages of the events within the respective gate.

**
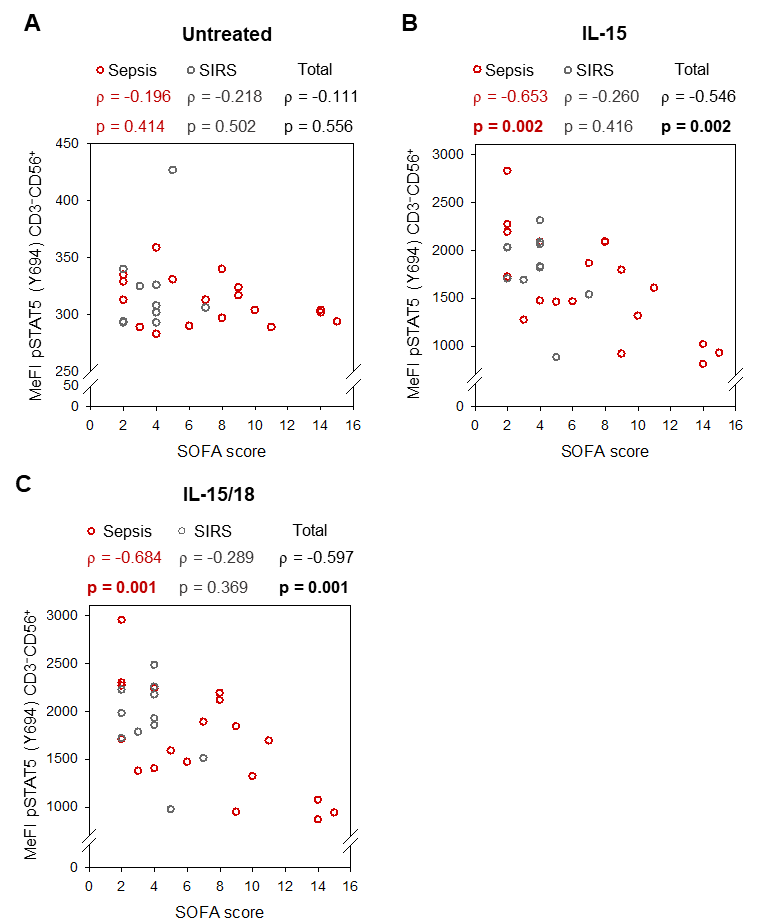
**

**Supplementary Figure 15. Correlations between SOFA score and pSTAT5 median fluorescence intensity (MeFI) values in sepsis and SIRS NK cells after cytokine treatment.** The correlations between SOFA and the MeFI values for STAT5 phosphorylated on Tyr694 (pSTAT5) in NK cells are plotted. Anticoagulated blood from patients with sepsis (red, n = 19) or SIRS (grey, n = 11) was left untreated (**A**) or incubated for 15 minutes at 37 °C with 45 ng/mL interleukin 15 (IL-15) (**B**) or IL-15 plus 50 ng/mL interleukin 18 (IL-15/18) (**C**). The values for Spearman’s ρ and for p are indicated for both patient subgroups separately and jointly (total).


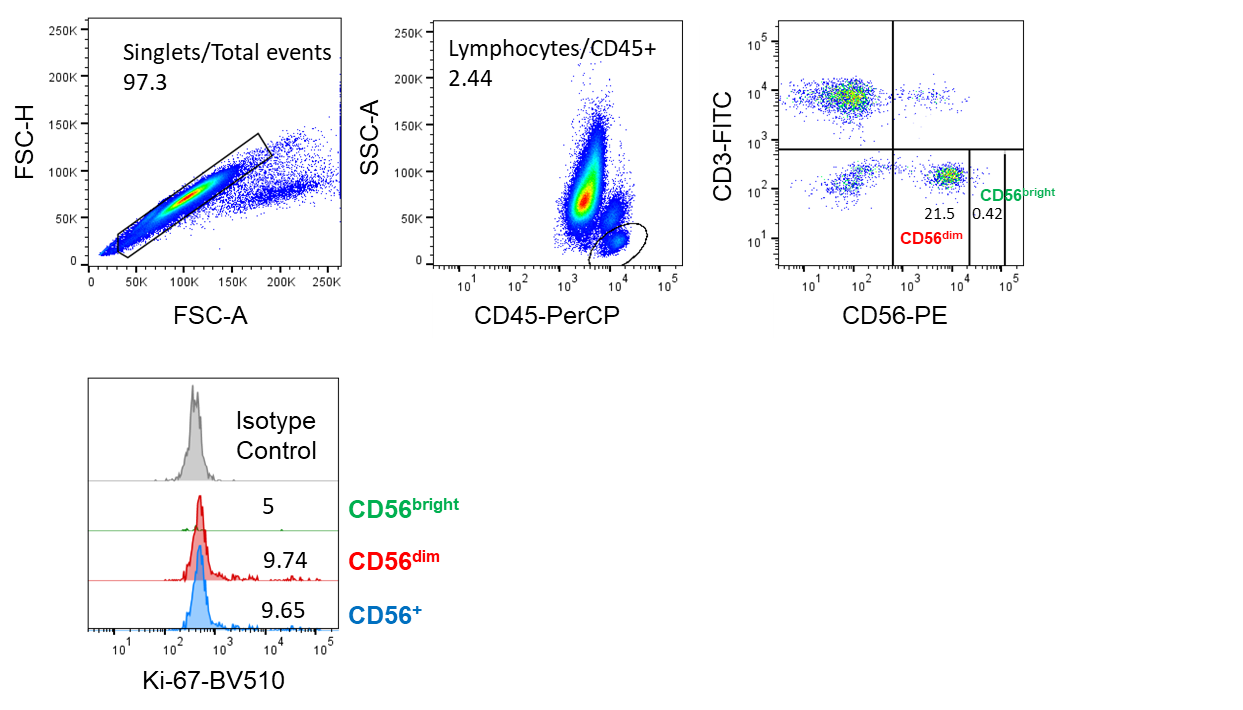


**Supplementary Figure 16. Representative flow cytometry analysis of Ki-67 in NK cell subsets.** Singlets were first gated based on FSC-A versus FSC-H, followed by selection of CD45⁺ lymphocytes, then CD3⁻CD56⁺ NK cells, distinguishing CD56^bright^ and CD56^dim^ subsets. Histograms show Ki-67 staining compared to isotype control in CD56^bright^, CD56^dim^, and total CD56⁺ NK cells. Numbers indicate percentages of the events within the respective gate.
